# Are psychosocial smoking cessation interventions delivered in pregnancy equally effective? A systematic review, meta-analysis and equity analysis of moderation analyses in randomized controlled trials

**DOI:** 10.1007/s10865-025-00614-6

**Published:** 2025-11-16

**Authors:** Claire Tatton, G. J. Melendez-Torres

**Affiliations:** Faculty of Health and Life Sciences, University of Exeter, Heavitree Road, Exeter, EX1 2LU UK

**Keywords:** Smoking in pregnancy, Systematic review, Equity

## Abstract

Rates of smoking in pregnancy have declined in most high-income countries. However, disparities between those of different socioeconomic statuses and ethnicities persist. We undertook this review to assess the effectiveness of interventions tailored to these equity-relevant characteristics on prenatal smoking cessation, postnatal abstinence and infant birth outcomes, and to assess whether outcomes are moderated by equity-relevant characteristics in universal interventions. We searched four databases and used supplementary search methods to identify randomized controlled trials of psychosocial smoking cessation interventions (including relapse prevention) delivered in pregnancy in high-income countries. We performed pairwise meta-analyses using robust variance estimation on tailored interventions and performed meta-regressions to test sensitivity to the proportion of minoritized ethnic participants in the trial sample. We analysed moderation of intervention effectiveness in universal interventions using harvest plots. We included 54 trials. Pairwise meta-analyses suggest intervention effectiveness for smoking cessation in pregnancy (OR 1.55, 95% CI 1.26, 1.91) and continued postnatal abstinence (OR 1.42 95% CI 1.19, 1.70). However, they did not suggest effectiveness for infant birth outcomes. In meta-regressions, effectiveness was moderated by the proportion of minoritized ethnic participants. Overall, prenatal smoking cessation and postnatal abstinence were not moderated by equity relevant characteristics in universal interventions. A reliance on universal interventions may be insufficient to positively affect existing disparities in smoking rates in pregnancy. Interventions that focus on addressing barriers to cessation related to material and financial deprivation may fail to account for broader sociocultural factors that are important to support minoritized ethnic groups.

**Supplementary Information:**

The online version contains supplementary material available at 10.1007/s10865-025-00614-6.

## Introduction

Reducing smoking during pregnancy is a continued public health priority due to the adverse outcomes on maternal and infant health, including increased risk of ectopic pregnancy, spontaneous miscarriage, stillbirth, preterm birth, and low birthweight (Bauld et al., 2017; Bonello et al., 2023). The impact of smoking in pregnancy also extends beyond birth to an increased risk of sudden infant death syndrome, asthma and obesity in childhood. Although smoking in pregnancy is a global issue, the prevalence is significantly higher in high-income countries compared to low- and middle-income countries [2]. Smoking is socially and economically patterned and is a significant contributor to health inequalities (Östergren, 2022). Those who smoke in pregnancy tend to have started smoking at a young age, have lower levels of educational attainment, work in lower wage occupations and are more likely to have partners, family members, or friends who also smoke (Bauld et al., 2017; Bonello et al., 2023). For those who try to stop smoking in pregnancy, a number of barriers exist. Increased nicotine metabolism in pregnancy can lead to exaggerated withdrawal symptoms, particularly for those with high nicotine dependence (Bowker et al., 2015; Griffiths et al., 2018). Additionally, the belief that smoking reduces stress, being exposed to the smoking of others, and fear of judgement and stigma from healthcare professionals can all have a negative impact on self-efficacy to quit (Bauld et al., 2017; Flemming et al., 2015; Fletcher et al., 2022; Tatton & Lloyd, 2023).

In most high-income countries, a reduction in rates of smoking in pregnancy has been observed since the 1990s. However, rates of decline have been slower in those of low socioeconomic status (SES) compared to high SES (Bonello et al., 2023). Additionally, differences in rates of smoking in pregnancy continue to be observed between different ethnic groups. Smoking prevalence in minoritized ethnic groups often reflect disparities in social and material deprivation (Azagba et al., 2020; Chamberlain et al., 2017; Washio & Cassey, 2016). This is observed in Aboriginal and Māori groups in Australia and New Zealand where rates are significantly higher than in non-Aboriginal and non-Māori people. However, this relationship is not consistently observed. For example, in the USA higher rates of smoking in pregnancy are observed in White women compared to African American, Hispanic and Asian‐Pacific women, but are similar between Alaskan Native and American Indian women and White women (Azagba et al., 2020; Chamberlain et al., 2017; Washio & Cassey, 2016). Whilst smoking rates vary between groups, the impact of tobacco use on maternal and infant health may be exacerbated by unequal access to and support from prenatal care experienced by minoritized ethnic groups, therefore contributing to health inequalities (Odd et al., 2024; Sheikh et al., 2022).

Findings from recent systematic reviews and meta-analyses suggest that individual psychosocial interventions can be effective for smoking cessation in pregnancy (Chamberlain et al., 2013, 2017; Griffiths et al., 2018; Heslehurst et al., 2020; Notley et al., 2025; Vila-Farinas et al., 2024) and for reducing rates of low birthweight (Chamberlain et al., 2013, 2017; Washio & Cassey, 2016). However, the continued effectiveness of interventions on postnatal abstinence from smoking and on reducing rates of preterm birth is mixed and less certain (Chamberlain et al., 2013, 2017; Heslehurst et al., 2020). Psychosocial interventions tend to work by incorporating features that affect attitudes and beliefs around smoking, address self-efficacy and motivation related to behavior change, take account of how social factors may impact personal beliefs and behaviors and help individuals to plan and prioritize action (Chamberlain et al., 2017). Whilst some of these reviews have examined differential effectiveness of interventions by equity-relevant characteristics by comparing pooled effects (Chamberlain et al., 2013, 2017; Vila-Farinas et al., 2024), assessment of equity impacts has not been conducted consistently in this area, or across a range of smoking and infant birth related outcomes. Therefore, we undertook this review to assess whether outcomes generated by psychosocial interventions are moderated by equity-relevant characteristics (socioeconomic status, race, and ethnicity). Understanding differences in intervention effectiveness is important to establish their impact on improving, or potentially worsening health inequalities.

The review questions are:

1. What is the effectiveness of interventions tailored to equity-relevant characteristics on prenatal smoking cessation, postnatal abstinence and infant birth outcomes?
2. Are prenatal smoking cessation, postnatal abstinence and infant birth outcomes, moderated by equity-relevant characteristics in universal interventions?

For this review we define tailored interventions as those which recruited only pregnant people of low socioeconomic status or minoritized ethnic groups to the trial sample, which delivered the intervention from a setting serving low socioeconomic status or minoritized ethnic groups or by intervention design characteristics. We define universal interventions as those aimed at those who smoke in pregnancy without further specific direction towards specific subgroups.

## Methods

### Registration and protocol

This review was registered with PROSPERO in January 2024 (CRD42024495353). As this was a systematic review, it did not require ethics approval.

### Eligibility criteria

We used the population, intervention, comparison, outcome and study design (PICOS) formula to design the eligibility criteria for this review. Studies were eligible for inclusion if the population were pregnant smokers living in high income countries (defined by The World Bank); regardless of age, years smoking, or nicotine dependence level. Trials that enrolled spontaneous quitters for relapse prevention support were also included. Relevant interventions were those delivered directly to smokers during pregnancy and those that continued to provide support postpartum. Intervention content was not restricted but could be generally classified as psychosocial or behavioral. Interventions could be single component—where one cessation strategy was employed, or multicomponent—where several strategies were offered. Multicomponent interventions could include access to pharmacotherapy; however, pharmacotherapy interventions alone were not eligible for inclusion. Interventions had to be either tailored to equity-relevant characteristics or be a universal intervention that included analysis of moderation effects by equity-relevant characteristics. We defined tailored interventions as those which had a trial recruitment strategy to recruit solely or majority low SES or minoritized ethnic participants, where the intervention was delivered in a setting or service accessed by these groups, or where the intervention had been designed with features relevant to these groups. Eligible comparators were usual care, waitlist, or other active psychosocial or behavioral interventions. The main outcome of interest was prenatal smoking cessation. Secondary outcomes of interest were postnatal abstinence from smoking and infant birth outcomes including birthweight, low birthweight, size for gestational age, APGAR score and preterm birth. Outcomes related to harm reduction or attitudinal changes were not eligible. Only randomized controlled trials (including cluster trials) were eligible for inclusion. The full inclusion and exclusion criteria are reported in Appendix 1.

### Information sources

We searched MEDLINE and PsycINFO via OvidSP, the Cochrane Central Register of Controlled Trials (CENTRAL) and the Cancer Research UK funded systematic review project of behavioral smoking cessation trials (IC-SMOKE database) for eligible studies. Searches were not restricted by date or language.

We reviewed the reference lists of systematic reviews retrieved through bibliographic database searching to identify any other relevant studies. Additionally, we used retrieved trial protocols to check that the associated outcome evaluations had also been retrieved. Where they had not, we searched clinical trial registries (ClinicalTrials.gov, WHO ICTRP, ANZCTR and ISRCTN) to identify the status of the trial and conducted further searching via Google Scholar by trial name, intervention name, or principal investigator name to locate published outcome evaluations. Finally, we performed citation searching on included studies using Scopus, CrossRef, Web of Science, and PubMed to identify any other eligible studies.

### Search, selection and data extraction

We developed the search strategy for MEDLINE and PsycINFO (via OvidSP) from the search strategy performed by Koch et al., (2019) in their systematic review on the effectiveness of smoking cessation interventions on disadvantaged socioeconomic position (Kock et al., 2019). We adapted the strategy for this review by including search terms for pregnant smokers and for race and ethnicity. We also removed the search terms for study design, and applied filters to limit the results by study design to randomized controlled trials only. The final searches were conducted in February 2024. The search strategies are reported in Appendix 1.

Results from searches conducted in all databases were exported to Endnote 21 for deduplication and review. Title and abstract and full text screening was undertaken by CT. GJMT independently reviewed 20% of the records at both title and abstract and full text screening stages (blinded). Judgements were compared, and disagreements settled through discussion. No retrievals were in non-English language and therefore no translation service was required. The data extraction template from a similar review (Melendez-Torres et al., 2023) was adapted for this review to collect data items regarding study sample, study design, participant characteristics including; age, ethnicity/race, tobacco dependence, and socioeconomic status, intervention characteristics, outcome measures including; instrument, biomarker and follow up timepoints, and tests of moderation. Where missing data within studies was expected to affect the analysis, we attempted to contact authors to request additional information. Data extraction was undertaken independently by CT and was quality assured by GJMT.

### Outcomes

For this review, prenatal smoking cessation was defined as non-smoking status registered at any timepoint during pregnancy, including at time of delivery. Postnatal abstinence from smoking was defined as non-smoking status registered at any time after childbirth. Measures of non-smoking status included self-reported, or self-reported with biochemical validation using cotinine sample (urine, saliva or serum) and/or exhaled carbon monoxide air. The biomarker levels used to define non-smoking status were taken from the included trials. Non-smoking status was defined as point prevalence or continuous abstinence. Infant birth outcomes were defined as mean birthweight, mean APGAR score, and rates of low birthweight, size for gestational age, and preterm birth.

### Synthesis strategy

Included studies were organized by intervention type (tailored or universal), category, number of components, and relevant outcomes. Interventions were categorized by their main strategy using the typology set out by Chamberlain et al., 2017 (Chamberlain et al., 2013) as shown in Table 1:

**Table 1:** intervention typology

Category	Definition
Counselling	Interventions focused on improving motivation to quit, increasing problem solving, and coping skills. Approaches may include motivational interviewing, cognitive behavior therapy, psychotherapy, or similar. These may be delivered in person or over the phone by a range of healthcare professionals or counsellors. For this review, we included materials which used personal testimonials and advice for quitting
Health education	Interventions that provided information on risks of smoking and advice to quit but offered no further support on how to do this. For this review, this included self-help booklets or other materials requiring self-directed use
Social support	Interventions where the support and encouragement of a peer, significant other, lay person, or professional was the main strategy to promote smoking cessation
Feedback	Interventions which provided feedback to the participant about the health of the fetus through ultrasound monitoring, carbon monoxide monitoring, or urine cotinine measurements. For this review, this did not include trials which used these methods to validate smoking status for the measurement of outcomes
Incentives	Interventions where participants received a financial incentive contingent on smoking behavior. Incentives could be received as vouchers, cash, or products. For this review, this did not include trials that provided participants with incentives for taking part in the trial
Single component	Interventions where only one cessation strategy was offered
Multi-component	Interventions where more than one strategy was offered, either other forms of psychosocial or behavioral support, or access to pharmacotherapy
Multi-level	Interventions which also included organizational or community level components to promote smoking cessation

### Pairwise meta-analysis

Meta-analyses of tailored interventions used random effects robust variance estimation (RVE). RVE improves on previous strategies for dealing with multiple relevant effect sizes within studies by allowing for inclusion of all relevant effect sizes whilst adjusting for interdependencies within studies, rather than requiring the selection of one effect size (Tanner-Smith & Tipton, 2014). Analyses were based on the intention to treat analysis reported in trials. Where this was unavailable, per protocol analysis was used instead. Pairwise meta-analyses compared each intervention against control and were grouped by outcome type and intervention type.

The key metric for meta-analyses of prenatal smoking cessation and postnatal smoking abstinence outcomes was the odds ratio. All included studies presented dichotomous outcomes for smoking status. Five trials included in the meta-analysis had no events in one or more of the trial arms (Bradizza et al., 2017; Glover et al., 2015; Lowe et al., 1998; Secker-Walker et al., 1997; Tuten et al., 2012). To avoid computational error, a fixed value of 0.5 was added to all cells to generate the odds ratio (Higgins JPT, 2023). The key metrics for meta-analyses of infant birth outcomes were the mean difference for birthweight and APGAR score, and odds ratios for rates of preterm birth and low birthweight. No included studies reported size for gestational age. We checked that cluster trials have taken account of any issues regarding the unit of analysis and combined these with participant level randomized trials within the meta-analyses. We included pooled effect sizes in forest plots. All meta-analyses were performed in Stata 18.

### Moderation analyses

We performed pairwise meta-regression for the primary outcome to test the relationship between the proportion of minority ethnic participants included in the trial sample and effectiveness. This was selected due to the differences in smoking in pregnancy rates observed between different ethnic groups and the inconsistent relationship observed between ethnicity, deprivation and smoking (Azagba et al., 2020; Chamberlain et al., 2017; Washio & Cassey, 2016). The proportion of minority ethnic participants was entered as both a continuous variable and a binary variable using a sample of 80% minoritized participants as a cut point.

Finally, we examined the equity impacts of universal interventions using moderator analyses undertaken in trials. Tailored interventions that presented a formal test of moderation were also examined. Data were organized by outcome and test for moderation, either by significance of an interaction term, or subgroup analysis. We used subgroup analyses to construct statistical estimates of effect modification with a standard z-test for equality of means. We constructed harvest plots to graphically depict the cumulative equity impacts of interventions. Individual bars represent outcome evaluations organized by intervention type and placed according to their moderation impact (e.g. where there is evidence suggesting a greater impact on one group, the other, or no gradient).

### Risk of bias and quality assessments

CT assessed risk of bias in all included trials using Cochrane Risk of Bias 2 Tool for Randomized Controlled Trials and Cluster Randomized Controlled Trials. GJMT independently assessed 10% of the records. Disagreements were settled through discussion. We assessed the quality of outcomes assessed in pairwise meta-analyses using the GRADE tool. We used precision-effect test and precision-effect estimate with standard errors (PET-PEESE) meta-regression to investigate possible publication bias to account for multiple dependent effect sizes. As there is no agreed appraisal tool for moderation analyses, we indicated where an indicative direction of moderation was used as opposed to a formal test as a marker of quality.

## Results

### Study characteristics

**Fig. 1:**
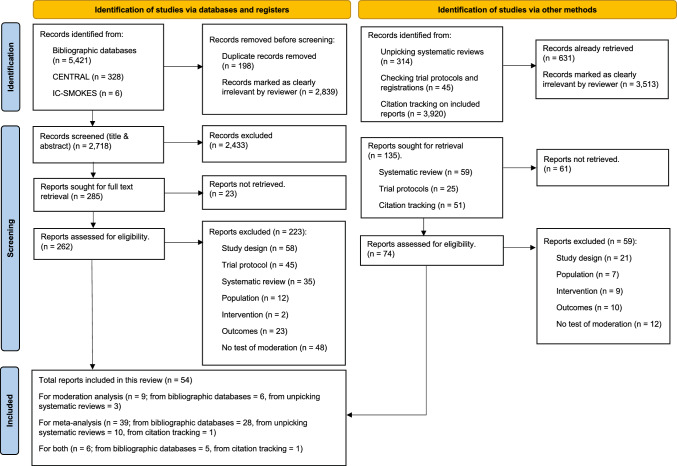
PRISMA flow diagram

The search and screening resulted in 54 outcome evaluations of 54 trials included in this review (Fig. 1). Full details of the characteristics of included trials are reported in Appendix 2.

Of the 54 outcome evaluations, six were of cluster randomized controlled trials (Hajek et al., 2001; Kendrick et al., 1995; Patten et al., 2020; Pbert et al., 2004; Polanska et al., 2005; Polańska et al., 2004) and 48 were of individually randomized controlled trials. The majority of trials (n = 42) were conducted in the USA. Four were conducted in Australia, (Eades et al., 2012; Lowe et al., 1998; Panjari et al., 1999) one in New Zealand, (Glover et al., 2015) one in The Netherlands, (Mejdoubi et al., 2014) one in Poland, (Polańska et al., 2004) four in the UK (Hajek et al., 2001; Lilley, 1986; Robling et al., 2016; Tappin et al., 2022) and one in Canada (Langford, 1983). The year of publication ranged from 1983 to 2022, and trial sample size ranged from 24 to 1120 participants.

Most trials (n = 26) had participants with a mean average age of < 26 years, 18 trials had participants with a mean average age of > 26 years and 10 trials did not report participants’ age. Most trials (n = 23) had participants with a mean gestational age within the second trimester (13–27 weeks pregnant), seven trials had participants with a mean gestational age within the first trimester (from conception to 12 weeks pregnant) (Eades et al., 2012; Klerman et al., 2001; Panjari et al., 1999; Solomon et al., 2000; Tappin et al., 2022; Windsor et al., 2000, 2011) and one trial had participants with a mean gestational age within the third trimester (28–40 weeks pregnant) (Reitzel et al., 2010). The remaining 23 trials did not report participant’s gestational age. In 21 trials, the trial sample was > 50% White participants and 21 studies had a trial sample of > 50% minoritized ethnic participants. Two trials had equal participation from White and minority ethnic participants (Baker et al., 2018) (Pbert et al., 2004) and 10 trials did not report the ethnicity of participants. Most trials (n = 32) had a sample size of > 50% participants of low SES (measured by household income, educational attainment, employment status, or health insurance). Seven trials had a sample size of > 50% high SES participants (Cinciripini et al., 2010; Hajek et al., 2001; Kendrick et al., 1995; Langford, 1983; Rigotti et al., 2006; Ruger et al., 2008; Tappin et al., 2022) and 6 trials had an equal sample of participants across SES (Abroms et al., 2017; Alaniz et al., 2019; Ershoff et al., 1999; Gielen et al., 1997; Lee et al., 2015; Reitzel et al., 2010). The remaining nine trials did not report SES of participants.

We included 45 trials tailored to equity relevant characteristics in pairwise meta-analysis. A further nine trials were not tailored, but conducted tests of moderation relevant to race, ethnicity or SES and were included in moderation analysis. Most (n = 29) were of counselling interventions, 14 were health education interventions, five were incentive interventions (Baker et al., 2018; Donatelle et al., 2000; Glover et al., 2015; Tappin et al., 2022; Tuten et al., 2012), four were social support interventions (Bullock et al., 2009; Hennrikus et al., 2010; Malchodi et al., 2003; Solomon et al., 2000), one was a feedback intervention (Patten et al., 2019) and one where the category of intervention was unclear (Hebel et al., 1985). Most (n = 41) were single component interventions, 12 were of multi-component interventions and two were multi-level interventions (Patten et al., 2020; Pbert et al., 2004). Trials of health education and counselling interventions were tested between 1983 and 2020 whereas trials of social support, incentives and feedback interventions were conducted from 2000 onwards.

### Quality / bias assessment

Only two trials (3.7%) were rated as low risk of bias (Abroms et al., 2017; Tappin et al., 2022), 29 trials (53.7%) were rated as some concerns of bias and 23 trials (42.6%) were rated as high risk of bias. The main sources of bias were unclear allocation concealment, inappropriate analyses used to estimate intervention effect that did not account for participants who left the trial after randomization, and measurement of the outcome where biochemical methods were not used to validate self-reported smoking status. Additionally, in cluster trials, the main source of bias was recruitment of participants after randomization of clusters without assurance that this did not affect participant selection.

GRADE assessments for pairwise meta-analyses are presented in Appendix 3. Prenatal smoking cessation was rated as very low certainty of evidence due to heterogeneity and borderline suspected publication bias (*p* = 0.046). Postnatal smoking abstinence was rated as low certainty due to heterogeneity, but publication bias was not suspected (*p* = 0.3). Infant birth outcomes were rated as moderate certainty of evidence. Funnel plots could not be constructed for infant birth outcomes due to the low number of studies included. Funnel plots for prenatal smoking cessation and postnatal abstinence are presented in Appendix 3.

### Summary statistics for relevant outcomes

The overall effect of the 45 trials included in pairwise meta-analyses are reported in Table 2 and forest plots are presented in Appendix 3. Of these, most (n = 35) were of interventions tailored to low SES (Abroms et al., 2017; Alaniz et al., 2019; Albrecht et al., 1998; Baker et al., 2018; Bradizza et al., 2017; Bullock et al., 2009; Burling, 1989; Cinciripini et al., 2010; Coleman-Cowger et al., 2018; Donatelle et al., 2000; Dornelas et al., 2006; Hennrikus et al., 2010; Kendrick et al., 1995; Lilley, 1986; Lowe et al., 1998; Malchodi et al., 2003; Mayer et al., 1990; Mejdoubi et al., 2014; Ondersma et al., 2012; Panjari et al., 1999; Pbert et al., 2004; Polanska et al., 2005; Polańska et al., 2004; Price et al., 1991; Reitzel et al., 2010; Robling et al., 2016; Ruger et al., 2008; Secker-Walker et al., 1998a, 1998b; Secker-Walker et al., 1997; Solomon et al., 2000; Stotts et al., 2004; Tuten et al., 2012; Windsor et al., 2011; Windsor et al., 2000), seven were tailored to minoritized ethnic groups (five of which were indigenous populations) (Eades et al., 2012; El-Mohandes et al., 2011; Glover et al., 2015; Klerman et al., 2001; Patten et al., 2010, 2019, 2020) and three were tailored to both characteristics (Forinash et al., 2018; Gielen et al., 1997; Lee et al., 2015). Of the 45 trials, 10 included spontaneous quitters in the trial sample. These outcomes were included in the meta-analyses.

**Table 2:** Pairwise meta-analyses

Outcome	k	No	OR (95% CI)	I^2^	Tau^2^
Prenatal smoking cessation	38	10,966	1.55 (1.26, 1.91) *	74%	0.24
Postnatal smoking abstinence	21	6891	1.43 (1.18, 1.73) *	53%	0.07
Low birthweight	4	882	0.56 (0.21, 0.92)	0%	0.00
Preterm birth	3	1289	0.52 (0, 1.04)	76%	0.18

Outcome	k	No	MD (95% CI)	I^**2**^	Tau^**2**^
Infant birthweight (grams)	7	3264	26.13 (− 12.98, 65.24)	0%	0.00
APGAR score	2	93	0.27 (− 0.20, 0.74)	0%	0.00

K, number of studies; No., number of participants; OR, odds ratio; CI, confidence interval; MD, mean difference

^*^Statistically significant effect size (*p* < 0.05)

Findings suggest that interventions were effective compared to controls for prenatal cessation and continued postnatal abstinence up to six months after childbirth. (Only two trials measured abstinence up to 12 months after childbirth Polanska et al., 2005; Secker-Walker et al., 1997)). However, heterogeneity was substantial. Findings for infant birth outcomes suggest that interventions were not effective compared to controls for all measures. No heterogeneity was observed for low birthweight, mean difference in birthweight and APGAR score, but was substantial for preterm birth.

### Heterogeneity

Pairwise meta-regressions (reported in Appendix 3) suggest that effectiveness for prenatal smoking cessation was moderated by proportion of minoritized ethnic participants in the trial sample. Trials with majority White participants were associated with effectiveness, but effectiveness decreased as the proportion of participants from minoritized ethnic groups (African American, Hispanic/Latina, Asian, American Indian, or Multiracial), increased. This finding was robust across meta-regressions entering proportion of minoritized participants as a continuous variable (OR 0.74 95% CI 0.36, 1.54) and as a binary variable distinguishing between trials with > 80% of minoritized participants and without (OR 0.81 95% CI 0.43, 1.52). Of trials (n = 6) with a majority sample of minoritized ethnic participants (> 80%), five were tailored to exclusively to Māori (n = 1) (Glover et al., 2015), Aboriginal or Torres Islanders (n = 1) (Eades et al., 2012), or Alaskan Natives (n = 3) participants (Patten et al., 2010, 2019, 2020), and one enrolled only African and American and White participants (Gielen et al., 1997).

### Moderation analysis

We constructed harvest plots to represent the moderation analyses included in 15 outcome evaluations (Abroms et al., 2017; Brandon et al., 2012; Ershoff et al., 1999; Forinash et al., 2018; Hajek et al., 2001; Hebel et al., 1985; Kendrick et al., 1995; Langford, 1983; Polańska et al., 2004; Reitzel et al., 2010; Rigotti et al., 2006; Robling et al., 2016; Strecher et al., 2000; Tappin et al., 2022; Windsor et al., 1993). Nine were of universal interventions, and six were of tailored interventions included in pairwise meta-analyses that also undertook a formal test of moderation. All 15 evaluations assessed moderation by socioeconomic status, whereas only seven assessed moderation by race or ethnicity (Abroms et al., 2017; Forinash et al., 2018; Hebel et al., 1985; Kendrick et al., 1995; Reitzel et al., 2010; Strecher et al., 2000; Windsor et al., 1993). In 11 evaluations, significance of an interaction term was reported, for three evaluations we constructed statistical estimates of effect modification from subgroups analyses, and for one evaluation we took the indicative direction of moderation from the subgroup analyses presented.

### Moderation of outcomes by socioeconomic status

One health education intervention demonstrated a pattern of moderation effects favoring those of low SES for postnatal smoking abstinence, but not all interaction tests were statistically significant (Brandon et al., 2012). Additionally, one counselling intervention demonstrated a pattern of moderation effects favoring those of low SES for prenatal smoking cessation, but not all interaction tests were statistically significant (Polańska et al., 2004). One counselling intervention demonstrated an indicative direction of moderation favoring participants of high SES for prenatal smoking cessation (Kendrick et al., 1995). One evaluation demonstrated no statistically significant difference between the birth weight of infants born to participants of low SES (Hebel et al., 1985). Nine evaluations only reported non-significance as opposed to moderation effects. Three were of counselling interventions; two for prenatal smoking cessation (Rigotti et al., 2006; Robling et al., 2016) and one to postnatal smoking abstinence (Reitzel et al., 2010). Five were of health education interventions; two for prenatal smoking cessation (Abroms et al., 2017; Ershoff et al., 1999), two for postnatal abstinence (Langford, 1983; Strecher et al., 2000) and one for both prenatal and postnatal smoking cessation (Hajek et al., 2001). One was of a financial incentive intervention for prenatal smoking cessation (Tappin et al., 2022).

**Fig. 2:**
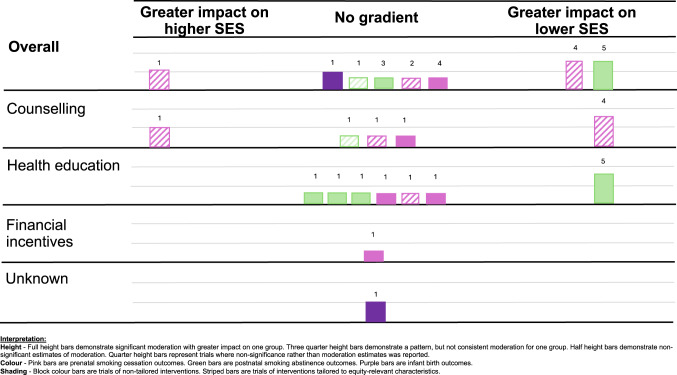
Harvest plot, socioeconomic status

Taking all outcomes together, the harvest plot (Fig. 2) does not provide evidence of a gradient in effectiveness of interventions according to SES.

### Moderation of outcomes by race and ethnicity

One counselling intervention demonstrated a statistically significant moderation effect favoring Black and Asian participants for prenatal smoking cessation (Forinash et al., 2018). One health education intervention suggested a moderation effect favoring non-White participants for prenatal smoking cessation, but this failed to reach significance (Windsor et al., 1993). One counselling intervention demonstrated an indicative direction of moderation favoring White participants for prenatal smoking cessation (Kendrick et al., 1995). One evaluation demonstrated no statistically significant difference between the birth weight of infants born to non-White participants (Hebel et al., 1985). The remaining three evaluations only reported non-significance as opposed to moderation effects, one health education intervention for prenatal smoking cessation (Abroms et al., 2017) and one health education intervention and one counselling intervention for postal smoking abstinence (Reitzel et al., 2010; Strecher et al., 2000).

**Fig. 3:**
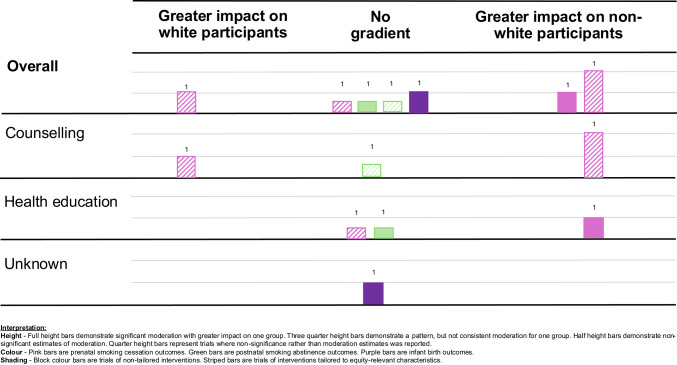
Harvest plot, race and ethnicity

Taking all outcomes together, the harvest plot (Fig. 3) does not provide evidence of a gradient in effectiveness of interventions according to race or ethnicity.

## Discussion

### Main findings

The aim of this review was to assess whether outcomes generated by individual, psychosocial interventions are moderated by equity-relevant characteristics, principally socioeconomic status (SES), race and ethnicity. The findings of our review suggest evidence for effectiveness of tailored interventions for prenatal smoking cessation. This finding is consistent with the results of other, recent systematic reviews (Chamberlain et al., 2013, 2017; Notley et al., 2025; Vila-Farinas et al., 2024). Our findings also suggest evidence for effectiveness of tailored interventions for continued postnatal abstinence from smoking, up to six months after childbirth. However, we did not find evidence for effectiveness of tailored interventions on improving infant birthweight or APGAR scores, or on decreasing the odds of low birthweight or preterm birth. Overall, evidence of moderation effects in interventions was sparse, with the majority of equity-relevant analyses reporting moderation effects related to SES. Of the outcome evaluations included in this review, findings suggest that neither prenatal or postnatal smoking outcomes were moderated by SES, race or ethnicity. There was insufficient evidence regarding moderation of infant birth outcomes. Whilst this offers some assurance that universal interventions do not appear to worsen health inequalities, it suggests that a reliance on universal interventions alone may be insufficient to positively affect existing disparities in smoking rates in pregnancy.

Additionally, this review highlights that existing trials tend to focus on intervention effectiveness for pregnant smokers of low SES compared to minoritized ethnic groups. A strength of this review is that we have studied intervention effectiveness by multiple equity-relevant characteristics. This acknowledges that the overrepresentation of minoritized groups by low SES means that for many, barriers to smoking cessation in pregnancy may be similar. However, our findings suggest that the effectiveness of tailored interventions was moderated by the proportion of minoritized ethnic participants in the sample. This highlights that a focus on perceived similarity of experience of material and financial deprivation may fail to take account of how multiple sources of oppression are experienced (Bowleg, 2012). Therefore, addressing broader sociocultural factors may be important to improving the access, experience, and outcomes of interventions for minoritized ethnic groups (Chamberlain et al., 2017; Washio & Cassey, 2016). This is especially important to addressing broader ethnic and racial disparities in prenatal outcomes and infant mortality rates (Burris & Parker, 2021; Odd et al., 2024; Sheikh et al., 2022).

Finally, our review highlights clear differences in the sufficiency of evidence regarding trials undertaken in different high-income countries and of different intervention types. The majority of trials included in this review were undertaken in the USA and were of counselling and health education interventions. Interest in financial incentive interventions for smoking cessation has grown considerably in recent years. These are thought to be particularly effective for those of low socioeconomic status as they can help meet financial needs which can alleviate stress, and increase autonomy and esteem (Notley et al., 2025). However, this review highlights there is currently limited available evidence regarding whether the effectiveness of financial incentives is experienced equally.

### Strengths and limitations

To our knowledge, this is the first review to focus on understanding the equity impacts of psychosocial smoking cessation interventions delivered in pregnancy across a broad set of smoking and infant birth outcomes. Using both meta-analysis and moderation analysis allowed us to comprehensively assess differences in effectiveness both between and within trials which, to our knowledge, has not previously been studied. Additionally, the use of robust variance estimation (RVE) meta-analysis is likely to have produced a more precise overall estimate of effectiveness than would have been achieved through selecting one effect size for each included trial in standard meta-analysis.

However, our review also has several limitations. First, whilst we undertook a range of search methods, we cannot exclude the possibility that eligible trials were missed. Second, incomplete reporting of moderation analyses likely restricted the number of trials that could be included in this review. It is expected that some exploratory analysis undertaken in trials was not reported, particularly where findings were non-significant or deemed to be unimportant. How this issue may have affected the findings presented in this review, however, cannot be estimated. Third, classification of interventions tailored to SES, ethnicity or both may have been inconsistent. We relied upon explicit reporting from trialists on how they had tailored interventions either through trial inclusion criteria, trial setting, or intervention design characteristics. It is possible that trialists’ knowledge of the demographic characteristics of the local area influenced how interventions were tailored to multiple characteristics and social identities that was not apparent to us. Fourth, the low number of trials of some intervention types and use of RVE meant we were unable to conduct subgroup analyses to assess differential effectiveness between intervention types. Fifth, meta-regressions relied on a small number of trials where the majority of participants were from minoritized ethnic groups. Finally, we were unable to examine all equity-relevant characteristics, therefore, our review is unable to contribute to understanding of how interventions are effective in addressing inequalities related to sexual orientation and gender-identity.

### Implications for policy and practice

The majority of tailored interventions included in this review were single component interventions. This suggests that delivering multiple cessation strategies, which may be more resource intensive and complicated to implement, may not be more effective than delivering a single cessation strategy. Several effective tailored interventions offered counselling that addressed relational and environmental stressors or used personal testimonials from peers to communicate the risks of smoking in pregnancy and advice for how to quit. Therefore, approaches that seek to understand and respond to individual circumstances and which utilize credible messengers with shared experience may increase the meaning and relevance of interventions to participants, optimizing them towards equity. However, the paucity of evidence in this review from outside the USA and for some intervention types means that practitioners should monitor interventions closely to ensure they are delivering equitable outcomes for their local population.

Future trials should report moderation effects to improve the understanding of equity impacts of interventions. Additionally, reviews that synthesize the characteristics and components of effective interventions for those of low SES and minoritized ethnic groups could provide explanatory accounts of equity promoting interventions for further testing through trials and could assist with shaping policy and practice guidelines.

## Conclusion

This review found that tailored psychosocial interventions demonstrated effectiveness for prenatal and postnatal smoking cessation, however, their impact on infant birth outcomes remains inconclusive. Additionally, we did not find evidence of moderation of effectiveness in universal interventions between participant groups defined by equity-relevant characteristics; however, this was based on a small number of trials. Finally, we found more evidence for interventions tailored to those of low SES than minoritized ethnic groups, and significant gaps in evidence across intervention types and from outside the USA. Together, these findings suggest that universal interventions alone may be insufficient to address disparities in smoking in pregnancy rates. Whilst tailored interventions show promise, this review highlights that they may currently fail to address sociocultural barriers to cessation experienced by minoritized ethnic groups. Finally, this review calls for improved reporting of moderation effects in trials, and improved reporting of intervention design features to generate a deeper understanding of relevant and inclusive cessation strategies.

## Supplementary Information

Below is the link to the electronic supplementary material.

Supplementary file1 (DOCX 17 kb)

Supplementary file2 (DOCX 141 kb)

Supplementary file3 (DOCX 2027 kb)

## Data Availability

All data generated or analysed during this study are included in this published article and its supplementary information files. Analytic code used to conduct the analyses presented in this study are not available in a public archive. They may be available by emailing the corresponding author. For the purpose of open access, the authors have applied a Creative Commons Attribution (CC BY) license to any Author Accepted Manuscript version arising from this submission.

## Code availability

Analytic code used to conduct the analyses presented in this study are not available in a public archive. They may be available by emailing the corresponding author.
