# Supplementary material for: Are psychosocial smoking cessation interventions delivered in pregnancy equally effective? A systematic review, meta-analysis and equity analysis of moderation analyses in randomized controlled trials
Source: J Behav Med. 2025 Nov 16;49(1):1–14. doi: 10.1007/s10865-025-00614-6 (PMC12945952; doi:10.1007/s10865-025-00614-6)
Supplement: Supplementary file 1 — Supplementary file1 (DOCX 17 kb) [file 10865_2025_614_MOESM1_ESM.docx]

Appendix 1 – eligibility criteria, search strategy and intervention typology

**Appendix 1 table 1 - Inclusion/exclusion criteria**

|  | **Inclusion** | **Exclusion** |
| --- | --- | --- |
| **Population** | Pregnant people living in high income countries who smoke in pregnancy without restriction of age, gestational age or nicotine dependence level.  People who spontaneously quit smoking upon becoming pregnant who enrol in interventions to remain abstinent.  People who smoke in pregnancy alongside other risk-taking behaviours (e.g. consuming alcohol, using substances) | Non-pregnant smokers  Pregnant people who smoke living in low- and middle-income countries. |
| **Intervention** | Psychosocial interventions delivered directly to women during pregnancy, including counselling, behavioural support, health or harm education, incentives, social support or exercise.  Interventions maybe delivered by a healthcare professional in a hospital setting or a community setting. Interventions maybe delivered by telephone or in a digital format (e.g. text, email, apps, websites, forums)  Interventions maybe single or multicomponent. Multicomponent interventions may include those that also incorporate access to pharmacological interventions.  Interventions maybe delivered as part of an integrated programme promoting healthy pregnancy or standalone.  Interventions maybe delivered during pregnancy only or both during pregnancy and after childbirth. | Pharmacotherapy only interventions  Hypnotherapy  Interventions delivered after childbirth only. |
| **Comparison** | Trials with a control condition, without specificity. Types of control may include usual care, a lower intensity intervention or an alternative intervention. | Trials with no control condition |
| **Outcomes** | **Primary outcome:**  1. Prenatal smoking cessation (non-smoking status registered at any time point during pregnancy, including time of delivery)  **Secondary outcomes:**  2. Postnatal abstinence (non-smoking status registered at any time after childbirth)  3. Birth outcomes (infant mean birthweight, size for gestational age, Apgar score or adverse birth events) | Partner/peer/family member smoking outcomes  Maternal mental health outcomes  Changes in attitude towards smoking, intention or confidence to change smoking behaviour  Harm reduction outcomes (reduction, quit attempts, time to relapse) |
| **Study designs** | All randomised controlled trial designs | All other study designs |
| **Date or language criteria** | All | None |
| **Subgroup analyses** | Tests for moderation by socioeconomic position (measured by education, household income, employment status, index of multiple deprivation or healthcare insurance status) and ethnicity (comparing majority populations compared to minority populations).  Tests maybe formal interaction tests or subgroup analyses. | No tests for moderation by equity relevant characteristics in non-tailored interventions. |

**Search strategies**

**Search strategy performed in OvidSP to search Medline and PsycINFO**

1. pregnan* or maternal ti,ab
2. smoking cessation or smok* quit* or smok* stop* or smok* cease or smok* cessat* or smok* give up ti,ab
3. behavio* or behavio?ral support or intervention or counsel* or brief or support or psychol* or individual* or individual-level or behavio?r therapy or cognitive therapy or target* or adapt* or tailor* not pharma* ti,ab
4. equity or equity impact or inequalit* or under-served or under served or underserved or marginali?ed or poor or affluent or disparit* or SES or socioeconomic or depriv* or disadvant* social class or occupation or employ or unemploy* or educat* or income or poverty or neighbo?r* or ethnic* or ethnic minorities or race or racial af

1 AND 2 AND 3 AND 4

Filter search results by clinical study or clinical trial, all or clinical trial or controlled clinical trial or pragmatic clinical trial or randomized controlled trial.

**Search strategy performed in Cochrane Central Register of Controlled Trials CENTRAL**

1. (smoke OR smoking OR tobacco):ti,ab,kw
2. (cessation OR stop* OR quit*):ti,ab,kw AND (pregnant OR pregnancy):ti,ab,kw
3. (behavio?r support OR counselling OR individual level):ti,ab,kw
4. #1 AND #2 AND #3 in Cochrane Central Register of Controlled Trials

All searches were performed in February 2024.
