# Supplementary material for: Are psychosocial smoking cessation interventions delivered in pregnancy equally effective? A systematic review, meta-analysis and equity analysis of moderation analyses in randomized controlled trials
Source: J Behav Med. 2025 Nov 16;49(1):1–14. doi: 10.1007/s10865-025-00614-6 (PMC12945952; doi:10.1007/s10865-025-00614-6)
Supplement: Supplementary file 2 — Supplementary file2 (DOCX 141 kb) [file 10865_2025_614_MOESM2_ESM.docx]

Appendix 2 – included studies

Table of Contents

[List of included studies 2](#_Toc172381473)

[Appendix 2 table 1 - study design of included studies 7](#_Toc172381474)

[Appendix 2 table 2 - intervention characteristics of included studies 12](#_Toc172381475)

[Appendix 2 table 3 - sample characteristics of included studies 37](#_Toc172381476)

[Appendix 2 table 4 - study level effects of included studies 51](#_Toc172381477)

# **List of included studies**

Abroms, Lorien C., Pamela R. Johnson, Leah E. Leavitt, Sean D. Cleary, Jessica Bushar, Thomas H. Brandon, and Shawn C. Chiang. 2017. 'A randomized trial of text messaging for smoking cessation in pregnant women', *American Journal of Preventive Medicine*, 53: 781-90.

Alaniz, K., B. Christiansen, T. Sullivan, L. Khalil, and M. C. Fiore. 2019. 'Addressing Postpartum Smoking Relapse Among Low-Income Women: a Randomized Control Trial', *Journal of patient-centered research and reviews*, 6: 233‐42.

Albrecht, S., L. Payne, C. A. Stone, and M. D. Reynolds. 1998. 'A preliminary study of the use of peer support in smoking cessation programs for pregnant adolescents', *J Am Acad Nurse Pract*, 10: 119-25.

Baker, T. B., D. L. Fraser, K. Kobinsky, R. Adsit, S. S. Smith, L. Khalil, K. M. Alaniz, T. E. Sullivan, M. L. Johnson, and M. C. Fiore. 2018. 'A randomized controlled trial of financial incentives to low income pregnant women to engage in smoking cessation treatment: effects on post-birth abstinence', *Journal of consulting and clinical psychology*, 86: 464‐73.

Bradizza, C. M., P. R. Stasiewicz, Y. Zhuo, M. Ruszczyk, S. A. Maisto, J. F. Lucke, T. H. Brandon, R. D. Eiden, K. S. Slosman, and P. Giarratano. 2017. 'Smoking Cessation for Pregnant Smokers: Development and Pilot Test of an Emotion Regulation Treatment Supplement to Standard Smoking Cessation for Negative Affect Smokers', *Nicotine & Tobacco Research*, 19: 578-84.

Brandon, T. H., V. N. Simmons, C. D. Meade, G. P. Quinn, E. N. Lopez Khoury, S. K. Sutton, and J. H. Lee. 2012. 'Self-help booklets for preventing postpartum smoking relapse: a randomized trial', *American journal of public health*, 102: 2109-15.

Bullock, L., K. D. Everett, P. D. Mullen, E. Geden, D. R. Longo, and R. Madsen. 2009. 'Baby BEEP: A randomized controlled trial of nurses' individualized social support for poor rural pregnant smokers', *Maternal & Child Health Journal*, 13: 395-406.

Burling T. A, Bigelow G. E, Robinson J. C, Mead A. M. 1989. 'Smoking during pregnancy: Reduction via objective assessment and directive advice', *Behaviour Therapy*, 22: 31-40.

Cinciripini, P. M., J. A. Blalock, J. A. Minnix, J. D. Robinson, V. L. Brown, C. Lam, D. W. Wetter, L. Schreindorfer, J. P. McCullough, P. Dolan-Mullen, A. L. Stotts, and M. Karam-Hage. 2010. 'Effects of an intensive depression-focused intervention for smoking cessation in pregnancy', *Journal of Consulting & Clinical Psychology*, 78: 44-54.

Coleman-Cowger, V. H., K. S. Mark, Z. R. Rosenberry, B. Koszowski, and M. Terplan. 2018. 'A Pilot Randomized Controlled Trial of a Phone-based Intervention for Smoking Cessation and Relapse Prevention in the Postpartum Period', *Journal of Addiction Medicine*, 12: 193-200.

Donatelle, R. J., S. L. Prows, D. Champeau, and D. Hudson. 2000. 'Randomised controlled trial using social support and financial incentives for high risk pregnant smokers: significant other supporter (SOS) program', *Tob Control*, 9 Suppl 3: III67-9.

Dornelas, E. A., J. Magnavita, T. Beazoglou, E. H. Fischer, C. Oncken, H. Lando, J. Greene, J. Barbagallo, R. Stepnowski, and E. Gregonis. 2006. 'Efficacy and cost-effectiveness of a clinic-based counseling intervention tested in an ethnically diverse sample of pregnant smokers', *Patient education and counseling*, 64: 342‐49.

Eades, S. J., R. W. Sanson-Fisher, M. Wenitong, K. Panaretto, C. D'Este, C. Gilligan, and J. Stewart. 2012. 'An intensive smoking intervention for pregnant Aboriginal and Torres Strait Islander women: a randomised controlled trial', *Medical Journal of Australia*, 197: 42‐46.

El-Mohandes, A. A., M. N. El-Khorazaty, M. Kiely, and M. G. Gantz. 2011. 'Smoking cessation and relapse among pregnant African-American smokers in Washington, DC', *Maternal & Child Health Journal*, 15 Suppl 1: S96-105.

Ershoff, D. H., V. P. Quinn, N. R. Boyd, J. Stern, M. Gregory, and D. Wirtschafter. 1999. 'The Kaiser Permanente prenatal smoking-cessation trial: when more isn't better, what is enough?', *American Journal of Preventive Medicine*, 17: 161‐68.

Forinash, A. B., A. Yancey, D. Chamness, J. Koerner, C. Inteso, C. Miller, G. Gross, and K. Mathews. 2018. 'Smoking Cessation Following Text Message Intervention in Pregnant Women', *Annals of Pharmacotherapy*, 52: 1109-16.

Gielen, A. C., R. Windsor, R. R. Faden, P. O'Campo, J. Repke, and M. Davis. 1997. 'Evaluation of a smoking cessation intervention for pregnant women in an urban prenatal clinic', *Health education research*, 12: 247‐54.

Glover, Marewa, Anette Kira, Natalie Walker, and Linda Bauld. 2015. 'Using incentives to encourage smoking abstinence among pregnant Indigenous women? A feasibility study', *Maternal and Child Health Journal*, 19: 1393-99.

Hajek, P., R. West, A. Lee, J. Foulds, L. Owen, J. R. Eiser, and N. Main. 2001. 'Randomized controlled trial of a midwife-delivered brief smoking cessation intervention in pregnancy', *Addiction (Abingdon, England)*, 96: 485‐94.

Hebel, J. R., P. Nowicki, and M. Sexton. 1985. 'The effect of antismoking intervention during pregnancy: an assessment of interactions with maternal characteristics', *Am J Epidemiol*, 122: 135-48.

Hennrikus, D., P. Pirie, W. Hellerstedt, H. A. Lando, J. Steele, and C. Dunn. 2010. 'Increasing support for smoking cessation during pregnancy and postpartum: results of a randomized controlled pilot study', *Prev Med*, 50: 134-7.

Kendrick, J. S., S. C. Zahniser, N. Miller, N. Salas, J. Stine, P. M. Gargiullo, R. L. Floyd, F. W. Spierto, M. Sexton, R. W. Metzger, and et al. 1995. 'Integrating smoking cessation into routine public prenatal care: the Smoking Cessation in Pregnancy project', *American journal of public health*, 85: 217-22.

Klerman, L. V., S. L. Ramey, R. L. Goldenberg, S. Marbury, J. Hou, and S. P. Cliver. 2001. 'A randomized trial of augmented prenatal care for multiple-risk, Medicaid-eligible African American women', *American journal of public health*, 91: 105‐11.

Langford, E. R, Thompson E. G, Tripp S. C. 1983. 'Smoking and Health Education During Pregnancy: Evaluation of a Program for Women in Prenatal Classes', *Canadian Journal of Public Health*, 74: 285-89.

Lee, M., S. M. Miller, K. Y. Wen, S. K. Hui, P. Roussi, and E. Hernandez. 2015. 'Cognitive-behavioral intervention to promote smoking cessation for pregnant and postpartum inner city women', *Journal of behavioral medicine*, 38: 932‐43.

Lilley J, Forster D. P. 1986. 'A randomised controlled trial of individual counsellingof smokers in pregnancy', *Public Health*, 100: 309-15.

Lowe, J. B., K. P. Balanda, and G. Clare. 1998. 'Evaluation of antenatal smoking cessation programs for pregnant women', *Australian and New Zealand journal of public health*, 22: 55‐59.

Malchodi, C. S., C. Oncken, E. A. Dornelas, L. Caramanica, E. Gregonis, and S. L. Curry. 2003. 'The effects of peer counseling on smoking cessation and reduction', *Obstet Gynecol*, 101: 504-10.

Mayer, J. P., B. Hawkins, and R. Todd. 1990. 'A randomized evaluation of smoking cessation interventions for pregnant women at a WIC clinic', *Am J Public Health*, 80: 76-8.

Mejdoubi, J., S. C. van den Heijkant, F. J. van Leerdam, M. Crone, A. Crijnen, and R. A. HiraSing. 2014. 'Effects of nurse home visitation on cigarette smoking, pregnancy outcomes and breastfeeding: a randomized controlled trial', *Midwifery*, 30: 688-95.

Ondersma, S. J., D. S. Svikis, P. K. Lam, V. S. Connors-Burge, D. M. Ledgerwood, and J. A. Hopper. 2012. 'A randomized trial of computer-delivered brief intervention and low-intensity contingency management for smoking during pregnancy', *Nicotine & Tobacco Research*, 14: 351-60.

Panjari, M., R. Bell, S. Bishop, J. Astbury, G. Rice, and J. Doery. 1999. 'A randomized controlled trial of a smoking cessation intervention during pregnancy', *Aust N Z J Obstet Gynaecol*, 39: 312-7.

Patten, C. A., K. R. Koller, C. A. Flanagan, V. Y. Hiratsuka, C. A. Hughes, A. W. Wolfe, P. A. Decker, K. Fruth, T. A. Brockman, M. Korpela, D. Gamez, C. Bronars, N. J. Murphy, D. Hatsukami, N. L. Benowitz, and T. K. Thomas. 2019. 'Biomarker feedback intervention for smoking cessation among Alaska Native pregnant women: Randomized pilot study', *Patient Education & Counseling*, 102: 528-35.

Patten, C. A., H. A. Lando, C. A. Desnoyers, M. J. Bock, L. Alexie, P. A. Decker, C. A. Hughes, K. Resnicow, L. Burhansstipanov, R. Boyer, and J. Klejka. 2020. 'Healthy Pregnancies Project: Cluster Randomized Controlled Trial of a Community Intervention to Reduce Tobacco Use among Alaska Native Women', *Int J Environ Res Public Health*, 17.

Patten, Christi A., Richard A. Windsor, Caroline C. Renner, Carrie Enoch, Angela Hochreiter, Caroline Nevak, Christina A. Smith, Paul A. Decker, Sarah Bonnema, Christine A. Hughes, and Tabetha Brockman. 2010. 'Feasibility of a tobacco cessation intervention for pregnant Alaska Native women', *Nicotine & Tobacco Research*, 12: 79-87.

Pbert, L., J. K. Ockene, J. Zapka, Y. Ma, K. V. Goins, C. Oncken, and A. M. Stoddard. 2004. 'A community health center smoking-cessation intervention for pregnant and postpartum women', *American Journal of Preventive Medicine*, 26: 377‐85.

Polańska, K., W. Hanke, W. Sobala, and J. B. Lowe. 2004. 'Efficacy and effectiveness of the smoking cessation program for pregnant women', *International journal of occupational medicine and environmental health*, 17: 369‐77.

Polanska, Kinga, Wojciech Hanke, and Wojciech Sobala. 2005. 'Smoking relapse one year after delivery among women who quit smoking during pregnancy', *International journal of occupational medicine and environmental health*, 18: 159-65.

Price, James H., Ronald A. Krol, Sharon M. Desmond, David P. Losh, Stephen M. Roberts, and Frank F. Snyder. 1991. 'Comparison of three antismoking interventions among pregnant women in an urban setting: A randomized trial.DP - Apr 1991', *Psychological Reports*, 68: 595-604.

Reitzel, L. R., J. I. Vidrine, M. S. Businelle, D. E. Kendzor, T. J. Costello, Y. Li, P. Daza, P. D. Mullen, M. M. Velasquez, P. M. Cinciripini, L. Cofta-Woerpel, and D. W. Wetter. 2010. 'Preventing postpartum smoking relapse among diverse low-income women: a randomized clinical trial', *Nicotine & Tobacco Research*, 12: 326-35.

Rigotti, N. A., E. R. Park, S. Regan, Y. Chang, K. Perry, B. Loudin, and V. Quinn. 2006. 'Efficacy of telephone counseling for pregnant smokers: a randomized controlled trial', *Obstetrics and gynecology*, 108: 83‐92.

Robling, M., M. J. Bekkers, K. Bell, C. C. Butler, R. Cannings-John, S. Channon, B. C. Martin, J. W. Gregory, K. Hood, A. Kemp, J. Kenkre, A. A. Montgomery, G. Moody, E. Owen-Jones, K. Pickett, G. Richardson, Z. E. Roberts, S. Ronaldson, J. Sanders, E. Stamuli, and D. Torgerson. 2016. 'Effectiveness of a nurse-led intensive home-visitation programme for first-time teenage mothers (Building Blocks): a pragmatic randomised controlled trial', *Lancet*, 387: 146-55.

Ruger, J. P., M. C. Weinstein, S. K. Hammond, M. H. Kearney, and K. M. Emmons. 2008. 'Cost-effectiveness of motivational interviewing for smoking cessation and relapse prevention among low-income pregnant women: a randomized controlled trial', *Value in health*, 11: 191‐98.

Secker-Walker, R. H., L. J. Solomon, B. S. Flynn, J. M. Skelly, and P. B. Mead. 1998a. 'Reducing smoking during pregnancy and postpartum: physician's advice supported by individual counseling', *Preventive Medicine*, 27: 422‐30.

———. 1998b. 'Smoking relapse prevention during pregnancy. A trial of coordinated advice from physicians and individual counseling', *American Journal of Preventive Medicine*, 15: 25‐31.

Secker-Walker, R. H., L. J. Solomon, B. M. Geller, B. S. Flynn, J. K. Worden, J. M. Skelly, and P. B. Mead. 1997. 'Modeling smoking cessation: exploring the use of a videotape to help pregnant women quit smoking', *Women & health*, 25: 23‐35.

Solomon, L. J., R. H. Secker-Walker, B. S. Flynn, J. M. Skelly, and E. L. Capeless. 2000. 'Proactive telephone peer support to help pregnant women stop smoking', *Tob Control*, 9 Suppl 3: III72-4.

Stotts, Angela L., Katherine A. DeLaune, Joy M. Schmitz, and John Grabowski. 2004. 'Impact of a motivational intervention on mechanisms of change in low-income pregnant smokers', *Addictive Behaviors*, 29: 1649-57.

Strecher, V. J., K. R. Bishop, J. Bernhardt, J. M. Thorp, B. Cheuvront, and P. Potts. 2000. 'Quit for keeps: tailored smoking cessation guides for pregnancy and beyond', *Tob Control*, 9 Suppl 3: III78-9.

Tappin, D., L. Sinclair, F. Kee, M. McFadden, L. Robinson-Smith, A. Mitchell, A. Keding, J. Watson, S. Watson, A. Dick, D. Torgerson, C. Hewitt, J. McKell, P. Hoddinott, F. M. Harris, K. A. Boyd, N. McMeekin, M. Ussher, and L. Bauld. 2022. 'Effect of financial voucher incentives provided with UK stop smoking services on the cessation of smoking in pregnant women (CPIT III): pragmatic, multicentre, single blinded, phase 3, randomised controlled trial', *BMJ*, 379: e071522.

Tuten, M., H. Fitzsimons, M. S. Chisolm, P. A. Nuzzo, and H. E. Jones. 2012. 'Contingent incentives reduce cigarette smoking among pregnant, methadone-maintained women: results of an initial feasibility and efficacy randomized clinical trial', *Addiction*, 107: 1868-77.

Windsor, R. A., J. B. Lowe, L. L. Perkins, D. Smith-Yoder, L. Artz, M. Crawford, K. Amburgy, and N. R. Boyd, Jr. 1993. 'Health education for pregnant smokers: its behavioral impact and cost benefit', *American journal of public health*, 83: 201-6.

Windsor, R. A., L. L. Woodby, T. M. Miller, J. M. Hardin, M. A. Crawford, and C. C. DiClemente. 2000. 'Effectiveness of Agency for Health Care Policy and Research clinical practice guideline and patient education methods for pregnant smokers in medicaid maternity care', *American journal of obstetrics and gynecology*, 182: 68‐75.

Windsor, R., L. Woodby, T. Miller, and M. Hardin. 2011. 'Effectiveness of Smoking Cessation and Reduction in Pregnancy Treatment (SCRIPT) methods in Medicaid-supported prenatal care: trial III', *Health education & behavior*, 38: 412‐22.

# **Appendix 2 table 1 - study design of included studies**

| **Author (year)** | **Intervention and comparison** | **Intervention type** | **Country** | **Study design** | **Enrolment start** | **Enrolment end** | **Total clusters** | **Total arms** | **Target population** |
| --- | --- | --- | --- | --- | --- | --- | --- | --- | --- |
| Abroms (2017) | Quit4Baby vs. Text4Baby | Tailored | USA | RCT | Jul-15 | Feb-16 | N/A | 2 | Smokers |
| Ershoff (1999) | Self-help booklet with midwife counselling calls vs. self-help booklet with computerised telephone support vs. Self-help booklet only | Not tailored | USA | RCT | Nov-96 | Jun-97 | N/A | 3 | Smokers |
| Forinash (2018) | SOC with text messaging vs. SOC only. | Tailored | USA | RCT | May-14 | Jan-16 | N/A | 2 | Smokers |
| Windsor (1993) | Supported and reinforced health education vs. self-directed health education | Not tailored | USA | RCT | Sep-87 | Nov-89 | N/A | 2 | Smokers |
| Alaniz (2019) | First Breath enhanced programme vs. First Breath programme | Tailored | USA | RCT | NR | NR | N/A | 2 | Smokers & spontaneous quitters |
| Baker (2018) | Enhanced financial incentives vs. routine financial incentives | Tailored | USA | RCT | Sep-12 | May-15 | N/A | 2 | Smokers & spontaneous quitters |
| Bullock (2008) | Social support + smoking cessation booklets vs. social support alone vs. booklets alone vs. smoking cessation pamphlet | Tailored | USA | RCT | Jan-02 | Oct-05 | N/A | 4 | Smokers |
| Coleman-Cowgear (2018) | Phone-based Postpartum Continuing Care vs. standard care | Tailored | USA | RCT | Mar-13 | Dec-13 | N/A | 2 | Smokers |
| Eades (2012) | Tailored advice and support vs. usual care | Tailored | Australia | RCT | Jun-05 | Dec-08 | N/A | 2 | Smokers & spontaneous quitters |
| Gielen (1997) | Health education and reinforcement vs. usual prenatal education | Tailored | USA | RCT | NR | NR | N/A | 2 | Smokers |
| Glover (2015) | Retail voucher vs. Products vs. usual care | Tailored | New Zealand | RCT | Dec-12 | Jun-13 | N/A | 3 | Smokers |
| Kendrick (1995) | Counselling and written information vs. usual care | Tailored | USA | cRCT | NR | NR | 3 | 2 | Smokers |
| Klerman (2011) | Augmented care vs. usual prenatal care | Tailored | USA | RCT | Mar-94 | Jun-96 | N/A | 2 | Smokers |
| Lee (2015) | Cognitive behavioural counselling vs. best practice care | Tailored | USA | RCT | Jan-03 | May-07 | N/A | 2 | Smokers |
| Lowe (1998) 1a | Counselling vs. brief advice | Tailored | Australia | RCT | Nov-91 | Jun-92 | N/A | 2 | Smokers |
| Lowe (1998) 1b | Healthy 2' booklet vs. brief advice | Tailored | Australia | RCT | Jan-93 | Mar-93 | N/A | 2 | Smokers |
| Mejdoubi (2014) | Voorzorg programme vs. V-MIS standard programme | Tailored | The Netherlands | RCT | 2007 | 2009 | N/A | 2 | Smokers |
| Patten (2019) | Biomarker feedback vs. Quit Tobacco Programme | Tailored | USA | RCT | Mar-15 | Jul-16 | N/A | 2 | Smokers |
| Patten (2010) | Multi-media education pack and counselling vs. usual care | Tailored | USA | RCT | 2007 | 2008 | N/A | 2 | Smokers |
| Pbert (2004) | Special intervention (smoking cessation programme and organisational screning) vs. usual care | Tailored | USA | cRCT | NR | NR | 5 | 2 | Smokers & spontaneous quitters |
| Polanska (2004) | Midwife delivered cessation programme vs. usual care | Tailored | Poland | cRCT | Dec-00 | Dec-01 | 15 | 2 | Smokers & spontaneous quitters |
| Polanska (2005) | Midwife delivered cessation programme vs. usual care | Tailored | Poland | cRCT | Dec-00 | Dec-01 | 15 | 2 | Smokers & spontaneous quitters |
| Price (1991) | Videotape vs. self-help booklet vs. Physicians advice | Tailored | USA | RCT | Dec-87 | Mar-89 | N/A | 3 | Smokers |
| Ruger (2008) | Motivational Interviewing vs. usual care | Tailored | USA | RCT | NR | NR | N/A | 2 | Smokers & spontaneous quitters |
| Tappin (2022) | Financial incentives vs. usual care | Not tailored | UK | RCT | Jan-18 | Apr-20 | N/A | 2 | Smokers |
| Windsor (2011) | SCRIPT procedures vs. usual care | Tailored | USA | RCT | NR | NR | N/A | 2 | Smokers |
| Windsor (2000) | SCRIPT procedures vs. usual care | Tailored | USA | RCT | Oct-97 | Jan-98 | N/A | 2 | Smokers |
| Stretcher (2000) | Tailored cessation guides vs. non-tailored cessation guides | Not tailored | USA | RCT | Dec-96 | Dec-97 | N/A | 2 | Smokers & spontaneous quitters |
| Donatelle (2000) | SOS program vs. usual care | Tailored | USA | RCT | Jun-96 | Jun-97 | N/A | 2 | Smokers |
| Langford (1983) | Prenatal classes with smoking cessation presentations vs. presentations with midwife follow up vs. standard prenatal classes | Not tailored | Canada | RCT | Oct-77 | Apr-78 | N/A | 3 | Smokers & spontaneous quitters |
| Mayer (1990) | Behaviour change counselling vs. risk information vs. usual smoking cessation information | Tailored | USA | RCT | Aug-85 | Aug-86 | N/A | 3 | Smokers |
| Panjari (1999) | Personalised care vs. usual care | Tailored | Australia | RCT | Apr-94 | Jun-96 | N/A | 2 | Smokers |
| Malchodi (2003) | Peer counselling vs. usual care | Tailored | USA | RCT | Jan-98 | Feb-00 | N/A | 2 | Smokers |
| Burling (1991) | Letter intervention vs. usual care | Tailored | USA | RCT | NR | NR | N/A | 2 | Smokers |
| Lilley (1986) | Individual anti-smoking advice vs. usual care | Tailored | UK | RCT | Mar-82 | May-82 | N/A | 2 | Smokers |
| Soloman (2000) | Phone peer support vs. usual care | Tailored | USA | RCT | 1996 | 1997 | N/A | 2 | Smokers |
| Patten (2020) | Peer counselling and community wide social marketing vs. usual care | Tailored | USA | cRCT | NR | NR | 16 | 2 | Smokers & spontaneous quitters |
| Robling (2015) | Family Nurse Partnership (FNP) Programme vs. usual care | Not tailored | UK | RCT | Jun-09 | Jul-10 | N/A | 2 | Smokers |
| Bradizza (2017) | Cognitive-Behavioral Smoking Cessation Treatment and Emotional Regulation Treatment vs Cognitive-Behavioral Smoking Cessation Treatment and Health and Lifestyle Intervention | Tailored | USA | RCT | Jul-09 | Jun-11 | N/A | 2 | Smokers |
| Dornelas (2006) | Smoking cessation counselling vs. usual care | Tailored | USA | RCT | NR | NR | N/A | 2 | Smokers |
| Secker-Walker (1997) | Videotape vs. usual care | Tailored | USA | RCT | Nov-92 | Apr-93 | N/A | 2 | Smokers |
| Hennrikus (2010) | Peer support vs. usual care | Tailored | USA | RCT | 2005 | 2005 | N/A | 2 | Smokers |
| Tuten (2012) | Contingent behavioural incentives vs. non-contingent behavioural incentives vs. treatment as usual | Tailored | USA | RCT | May-05 | Jan-09 | N/A | 3 | Smokers |
| Brandon (2012) | Forever Free booklets vs. usual care | Not tailored | USA | RCT | Apr-04 | Apr-07 | N/A | 2 | Smokers & spontaneous quitters |
| Hajek (2001) | Midwife advice vs. standard smoking cessation information | Not tailored | UK | cRCT | NR | NR | NR | 2 | Smokers & spontaneous quitters |
| Secker-Walker (1998) 1a | Structured advice and individual counselling vs. usual care | Tailored | USA | RCT | Oct-88 | Oct-92 | N/A | 2 | Smokers |
| Secker-Walker (1998) 1b | Structured advice and individual counselling vs. usual care | Tailored | USA | RCT | Oct-88 | Oct-92 | N/A | 2 | Smokers & spontaneous quitters |
| Reitzel (2010) | MAPS vs. MAPS+ vs. self-help materials | Tailored | USA | RCT | Oct-04 | Apr-08 | N/A | 3 | Smokers & spontaneous quitters |
| Ondersma (2011) | CD5As vs. CM- Lite vs. Combined CD5As + CM-Lite vs. usual care | Tailored | USA | RCT | Jul-08 | Nov-09 | N/A | 4 | Smokers |
| Hebel (1985) | Treatment vs. control | Not tailored | USA | RCT | NR | NR | N/A | 2 | Smokers |
| Albrecht (1998) | Teen Freshstart vs. Teen Freshstart plus buddy vs. usual care | Tailored | USA | RCT | NR | NR | N/A | 3 | Smokers |
| Stotts (2004) | Motivational interviewing vs. usual care | Tailored | USA | RCT | NR | NR | N/A | 2 | Smokers |
| El-Mohandes (2011) | Smoking cessation intervention vs. usual care | Tailored | USA | RCT | Jul-01 | Oct-03 | N/A | 2 | Smokers & spontaneous quitters |
| Rigotti (2006) | Counselling vs. 'best practice' | Not tailored | USA | RCT | Sep-01 | Jun-04 | N/A | 2 | Smokers |
| Cinciripini (2010) | Cognitive Behavioral Analysis System of Psychotherapy vs. Health and Wellness | Tailored | USA | RCT | Jan-05 | Jan-08 | N/A | 2 | Smokers |

*NR = not reported, N/A = not applicable, RCT = randomized controlled trial, cRCT = cluster randomized trial*

# **Appendix 2 table 2 - intervention characteristics of included studies**

| **Author (year)** | **Intervention name** | **Intervention category** | **Components** | **Intervention description** | **Facilitator** | **Mode** | **Duration of intervention** |
| --- | --- | --- | --- | --- | --- | --- | --- |
| Abroms (2017) | Quit4Baby | Health education | Single | Messages aimed at improving self-efficacy and capability quit, describing the outcome expectations both to the mother and baby from quitting and increasing social support for quitting via an ex-smoker ‘quit pal’. Texts are timed around enrolment date, the quit date, and the baby’s due date. Highest number of messages sent around quit date and taper over time and stop after 3 months, except for monthly smoking-status program surveys that continue for 6 months. Additionally, 24 messages related to postpartum relapse prevention were delivered between 1 month prior to due date and 6 months after childbirth. | No facilitator | Digital | Messages from enrolment to 6 months after due date |
| Ershoff (1999) | Self-help booklet with midwife counselling calls | Health education | Single | Includes advice about preparing to quit, setting a quit date, methods for quitting, obtaining social support, and relapse-prevention strategies. Advice about exercise, diet, and stress management are also included. | No facilitator | Self-support | NR |
| Forinash (2018) | Standard of care (SOC) smoking cessation programme with text messaging | Counselling | Multiple | In addition to standard of care smoking in pregnancy programme, motivational text messages focused on smoking cessation and pregnancy. Sent 3 days prior to the quit date, 1 day prior, on their quit date, and then continued in de-escalating fashion until delivery. Messages also included reminders to refill medication. | No facilitator | Digital | From 3 days prior to quit date through to childbirth |
| Windsor (1993) | Supported and reinforced health education | Health education | Multiple | 15-minute intervention with trained health counsellor at first prenatal appointment about smoking cessation, risks and taught how to use the 7-day self-directed cessation guide. Cessation reinforcement included in medical records and letters. Finally, social support methods through newsletter of other testimonials, letters from a buddy. | Counsellor | Unclear | NR |
| Alaniz (2019) | First Breath enhanced smoking cessation programme | Counselling | Multiple | Added elements to increase the intensity of the First Breath program: additional counselling delivered postpartum (both telephonically and during home visits), CO monitoring, incentives for accepting home visits and for abstinence, and education/support to other household members. | Counsellor | In person | NR |
| Baker (2018) | Enhanced financial incentives | Incentives | Single | $40 enrolment pre-birth, 6x $25 pre-birth provider visits, 2 x $25 post-birth home visits, 5 x $20 post-birth phone calls, 2x $40 for taking CO tests with an additional $40 if CO tests passed. Max. possible incentives = $500 | Other HCP | Combination | From 1st trimester to 6 months post-birth. |
| Bullock (2008) | Baby BEEP programme | Social support | Single | Scheduled weekly telephone call and 24-h access to the nurse for any additional social support needed in relation to pregnancy including support in response to manage stress. Plus 8 booklets comprising a stop smoking for pregnant women. First provided at recruitment and remaining 7 posted at weekly intervals. | Nurse | Combination | Unclear |
| Coleman-Cowgear (2018) | Phone-based Postpartum continuing care | Counselling | Single | 10 proactive calls beginning in the third trimester of pregnancy and continuing through 6 months postpartum with the option for participants to call in to a 24/7 toll-free number, different from the standard referral state quit line, in the event of craving, lapse, or relapse. Standard care available in prenatal period. | Other HCP | Telephone | From week 36 of pregnancy to 6 months postpartum |
| Eades (2012) | Tailored cessation advice and support | Health education | Multiple | Support from GP and healthcare workers, support to partner/supporter and access to NRT if needed (after two failed attempts to quit) | GP | In person | Follow-up visits scheduled 3–5 days and 7–10 days after first antenatal visit |
| Gielen (1997) | Health education and reinforcement | Health education | Multiple | Guide to quitting smoking in pregnancy, 15 min counselling with peer health educator to help use the booklet, educational materials for cessation support, clinic reinforcement (both verbal and written) from RN and MD. | Other HCP | In person | First 3 components delivered at first prenatal appointment, 4th (reinforcement) at every prenatal appointment |
| Glover (2015) | Retail voucher incentives | Incentives | Multiple | Usual cessation support plus a retail voucher to the value of NZ$25 for each ‘abstinent from smoking’ week for eight weeks (up to NZ$200 max). Access to cessation information and NRT (usual care). | No facilitator | Unclear | 8 weeks from enrolment |
| Kendrick (1995) | Counselling and smoking cessation information | Counselling | Single | A protocol for 1-to-5-minute counselling sessions with the following minimum components: assessment of smoking status, discussion of quitting tips, and a supportive statement by the nurse clinician. A similar but abbreviated protocol was followed at WIC sites. The printed materials included a health care provider's guide to the smoking cessation protocols, eight brochures for pregnant smokers, and a brochure for postpartum women | Unclear | In person | NR |
| Klerman (2011) | Augmented prenatal care and advice with smoking cessation support | Counselling | Multiple | A structured smoking cessation/reduction program, adapted from Ershoff et al. (1989) was included in the programme. This provided written information on harms of smoking in pregnancy and how to stop, support from a health educator, individualized follow-up activities, logs, and technical assistance from a behavioural medicine specialist. Broader augmented care programme also included building social support from women's families and friends | Nurse | In person | Appointments every 2 weeks (40 mins) until last month of pregnancy where appointments were weekly |
| Lee (2015) | Cognitive behavioural counselling | Counselling | Single | 4 sessions with a health educator (2 prenatal, 2 postpartum). Sessions were designed to identify and address participants’ cognitive-affective barriers to smoking cessation focusing on risk perceptions, quitting self-efficacy, pros and cons of quitting, emotional distress, and self-regulatory strategies. An individual’s particular barriers to cessation were identified by her cognitive-affective responses elicited through prompts and role-play exercises during the initial session | Counsellor | Combination | Prenatal sessions = 13–25 weeks and 26–38 weeks’ gestation. Postnatal sessions = 2-6 weeks and 8-10 weeks postpartum. Final session by phone |
| Lowe (1998) 1a | Smoking cessation counselling | Counselling | Single | 15 min counselling session on the harms of smoking and how to use the booklet. Social contracts of support signed between pregnant smokers and partner/supporter. Access to brief advice intervention also. | Midwife | In person | NR |
| Lowe (1998) 1b | Healthy 2 Education booklet | Health education | Single | Booklet adapted to the feedback of focus group from trial 1. | No facilitator | Self-support | NR |
| Mejdoubi (2014) | Voorzorg Nurse cessation programme | Counselling | Single | V-MIS programme delivered at home visits by specialist nurses. Programme delivered alongside breastfeeding promotion. After birth, the VoorZorg nurses focused on the negative health effects for the baby and to refrain from smoking in the presence of the baby, | Nurse | In person | 10 home visits in pregnancy, 20 in the first year following childbirth and 20 visits in the second year |
| Patten (2019) | Biomarker feedback | Feedback | Multiple | Brochure describing the cotinine results, graphically illustrating the correlation between maternal urine cotinine concentrations and neonatal urine NNAL levels to provide feedback on their baby’s likely exposure to NNAL. During the three study calls, as part of the 5A’s, the counsellor reviewed the biomarker data and emphasized tobacco exposure risks to mother and baby, using motivational interviewing. The participant was asked to discuss her interpretation of her personalized cotinine results including assessment of her thoughts, feelings, and reactions to the information and perceived impact on current cigarette use. Reinforcement of behaviour change by providing information on how smoking cessation will reduce harmful consequences. To enhance self-efficacy, counsellors assisted participants to set short term goals. | Counsellor | Telephone | Three study calls lasting approximately 10–20 min each occurred at weeks 2, 3, and 4 after enrolment. Women received calls until 1 year postpartum, also. |
| Patten (2010) | Multi-media education pack and counselling | Counselling | Single | Patient education methods (video, cessation guide, telephone counselling) were adapted from the SCRIPT trials (Windsor, 1999). At the first visit, participants in this condition received the cessation guide and 15–25 min of counselling based on the recommended 5 A’s. Next, the woman viewed the video in private. The counsellor then spent 10–15 min discussing the video, teaching cessation skills, emphasizing the importance of establishing tobacco-free homes and families, and problem solving of potential barriers to enhance the woman’s self-efficacy to quit tobacco. The video was provided to the woman for in-home viewing with family members to elicit their support. Participants were scheduled for four 10–15 min proactive telephone sessions, at Weeks 1, 2, 4, and 6. At each session, the counsellor reviewed the participant’s tobacco use and assessed motivation level and self-efficacy to quit. | Counsellor | Combination | Weeks 1-6 after enrolment |
| Pbert (2004) | Smoking cessation 'special' intervention | Health education | Multi-level | (1) provider training to deliver a smoking intervention based on national clinical practice guidelines tailored to the woman's stage of change and delivered through three channels (obstetric, paediatric, and WIC providers); (2) an office practice management system to routinely screen for smoking status, prompt/remind providers to intervene, document the encounter, distribute materials, and arrange follow-up; and (3) establishment of program boards to coordinate the transfer of documentation among clinics, including periodic meetings with representatives from all clinics. | Unclear | Unclear | NR |
| Polanska (2004) | Midwife smoking cessation programme | Counselling | Single | Four home visits from the midwife. The first visit started with a diagnosis of the level of smoking addiction, using the Fagerström method, which measures physical dependence on nicotine. The visit continued with a discussion on the benefits of smoking cessation. During the second visit, about 1–2 weeks later, the pregnant woman who decided to give up smoking determined when this was to be done and signed the “Declaration to quit smoking.” On the third visit, scheduled 1–2 days after the designated quitting day, the midwife inquired whether the woman had actually quit smoking as she promised. On the fourth visit, one month after the quitting day, the midwife informed the woman how to avoid smoking and maintain smoking abstinence. | Midwife | In person | Approx. 2 months |
| Polanska (2005) | Midwife smoking cessation programme | Counselling | Single | Four home visits from the midwife. The first visit started with a diagnosis of the level of smoking addiction, using the Fagerström method, which measures physical dependence on nicotine. The visit continued with a discussion on the benefits of smoking cessation. During the second visit, about 1–2 weeks later, the pregnant woman who decided to give up smoking determined when this was to be done and signed the “Declaration to quit smoking.” On the third visit, scheduled 1–2 days after the designated quitting day, the midwife inquired whether the woman had actually quit smoking as she promised. On the fourth visit, one month after the quitting day, the midwife informed the woman how to avoid smoking and maintain smoking abstinence. | Midwife | In person | Approx. 2 months |
| Price (1991) | Videotape cessation advice | Health education | Single | 6.5 min videotape depicting risks of smoking in pregnancy and benefits to baby from quitting immediately. Pamphlet on how to quit smoking and were opportunity to ask questions of the health educator. Second videotape (4 min.) 1 month later highlighting the information on the previous videotape and then focusing on a variety of strategies to quit smoking. Opportunity to ask questions of the health educator | Unclear | In person | Initial appointment and 1 month follow up |
| Ruger (2008) | Motivational interviewing | Counselling | Single | Average of three home visits to deliver MI sessions: 1) educated clients about the impact of smoking on mothers, foetuses, and new-borns; 2) helped clients evaluate their smoking behaviour; 3) helped increase self-efficacy for smoking cessation and abstinence; 4) provided information on reducing exposure to environmental tobacco smoke and set goals on changes in smoking; and 5) provided feedback about household nicotine levels. The MI components were tailored to each client’s stage of readiness and MI sessions lasted 1 hour on average. MI subjects also received self-help smoking cessation manuals. | Unclear | In person | NR |
| Tappin (2022) | Financial incentives | Incentives | Multiple | Intervention participants were offered the addition of up to £400 at four timepoints. Firstly, a £50 voucher to engage with stop smoking services and set a stop smoking date. Secondly, if a stop smoking date was set, a £50 voucher if not smoking after four weeks confirmed by carbon monoxide. Thirdly, if abstinent at four weeks, a £100 voucher if smoke-free after 12 weeks confirmed by carbon monoxide. Fourthly, for all intervention participants, a £200 voucher if carbon monoxide verified smokefree, when the call centre phoned in late pregnancy at a random date between 34- and 38-weeks’ gestation. Incentives were LoveToShop shopping vouchers redeemable in many retail outlets. Access to control intervention also provided | No facilitator | Combination | From enrolment to 34-38 weeks’ gestation |
| Windsor (2011) | SCRIPT procedures | Counselling | Single | 4/5As (Ask–Advise–Assess–Arrange) Procedures 1, 2, 3, 9 and 10. Plus (Assist) Procedures 4 through 8 - (1) A Commit to Quit Smoking During and After Pregnancy video (2) A Pregnant Woman’s Guide to Quit Smoking, and (3) A ≤10-minute counselling session | Unclear | In person | NR |
| Windsor (2000) | SCRIPT procedures | Counselling | Single | In addition to the ask and advise components, experimental group patients were provided a 3-component patient education program (assist and arrange) (1) A Commit to Quit Smoking During and After Pregnancy video (2) A Pregnant Woman’s Guide to Quit Smoking, and (3) A ≤5-minute counselling session to increase motivation and to help each patient prepare a personal action plan to quit smoking | Unclear | In person | NR |
| Stretcher (2000) | Tailored smoking cessation guides | Health education | Single | Self-help guide with behaviour change messages tailored to participant responses provided on behavioural and psychosocial variables, including: stages of change for quitting smoking, perceived benefits of and barriers to quitting, self-efficacy, and demographic information. Materials were mailed after each prenatal visit where the questionnaire was completed | No facilitator | Self-support | NR |
| Donatelle (2000) | Significant Other Supporter (SOS) programme | Incentives | Multiple | Self-help kit - A pregnant woman’s guide to quit smoking, financial incentives for smoking abstinence, social support from designated supporter (preferably female, non-smoker) with financial incentives for supporter also for pregnant women’s smoking abstinence. Financial incentive vouchers worth $50.00/ month for confirmed quitters each month through two months postpartum. Where social support was provided the social supporter of successful treatment quitters received $50.00 voucher the first quit month, $25.00 the additional quit months, and $50.00 the last quit month. | Other HCP | Combination | From enrolment to two months postpartum |
| Langford (1983) | Prenatal classes with smoking cessation presentations | Health education | Single | Received a half hour presentation and pamphlet on smoking and pregnancy in the second prenatal class of the eight-week series | Nurse | In person | Eight-week prenatal classes |
| Mayer (1990) | Behaviour change counselling | Counselling | Single | a 20-minute one-to-one counselling session including both risk information and behaviour change components. RI component - "Because I Love My Baby" materials developed by the American Lung Association. These materials included a 'flip chart" presented by a health educator and provided in a printed brochure for clients to take home. BC component - self-help manual developed from Windsor, 1985 and the American Lung Association's "Freedom from Smoking program." An individual behavioural contract was developed specifying a quit date and selected a significant other as a cosigner. Self-monitoring included charts for recording daily smoking behaviour, and the development of an individualized plan of action for breaking recorded behavioural chains. | Unclear | In person | NR |
| Panjari (1999) | Personalised care | Counselling | Single | Usual care plus 4 counselling sessions from a smoking cessation trained midwife. Session one at the first visit ran for about 25 minutes, was multifaceted and based on cognitive therapy. The session consisted of the distribution of Quit literature; viewing a video followed by a discussion of its contents; delivery of a strong verbal message about the risks associated with smoking in pregnancy and advice to quit. The counselling component included concepts such as identification of smoking cues, discussion of the costs and benefits of quitting, and goal setting. Counselling sessions were personalized, informal and nonjudgemental. Emotional needs, especially feelings of guilt, were frequently discussed. Subsequent sessions, usually 5- 10 minutes in length, consisted of personalized counselling and distribution of literature as appropriate for each patient’s progress. Follow-up sessions were offered at around 16-20, 24- and 28-weeks’ gestation | Midwife | In person | To 28 weeks’ gestation |
| Malchodi (2003) | Peer counselling | Social support | Single | Usual care provided by HCPs. Additionally, subjects were assigned a peer counsellor. Peer counsellors were instructed to encourage the pregnant woman in the quit attempt, communicate caring and concern, encourage the woman to talk about the quitting process, and to reinforce basic information about smoking and successful quitting. Each counsellor was instructed to have eight client contacts with each participant. During this client contact, the peer counsellor supported and reinforced the stop smoking messages of the health care providers using a standardized prompt sheet. Peer counsellors were instructed to document the date, location, type, and length of each visit, as well as capture the reported stage of change and describe the peer counsellor and mother interaction. | Other HCP | Combination | Eight sessions during prenatal period |
| Burling (1991) | Physician letter intervention | Health education | Single | Standard clinic program plus a personal letter from the Chief of the Prenatal Clinic within 3 days of their first smoking assessment. Letter was directive, identifying the woman as a smoker and urging her to quit. An American Cancer Society pamphlet on smoking during pregnancy, "Why Start a Life Under a Cloud?" (American Cancer Society, 1982), was also included to describe in more detail the negative effects of smoking during pregnancy and to offer simple guidelines on self-directed smoking cessation efforts. | Nurse | Combination | NR |
| Lilley (1986) | Individualised anti-smoking advice | Health education | Single | In addition to usual care and advice, participants received individual anti-smoking advice, last approx. 10 mins from the SHO at the Hospital at the time of the initial interview. booklet A. Copies of this booklet, plus another Health Education Council leaflet entitled 'How to stop smoking, for you and your baby', were given to these women. Additional copies of booklet A were given to the patients specifically for partners who smoked or any other household members who smoked. They were advised that they would be followed up at home at 4 weeks (a provisional date was given) to further discuss smoking. Two weeks after the initial interview a letter reinforcing advice and giving additional encouragement was sent. Two weeks after this, the home visit was made. If the patient was at home further personal advice was given. If the patient was not at home, a standard letter including anti-smoking advice was left. The GP of each test group patient was informed of the survey by means of a letter identifying the individual included in the trial, explaining the purpose of the study, and including a copy of the main booklet used. They were requested to reinforce anti-smoking advice during their usual contact with the patient. | GP | Combination | To 4 weeks after initial appointment |
| Soloman (2000) | Phone-based peer support | Social support | Single | Same advice and materials as those receiving usual care. Plus, the offer of telephone peer support for women with moderate or high intentions of quitting smoking during their pregnancy. The brief counselling session (45 min) legitimised concern about smoking, elicited feelings about quitting, and encouraged progress toward change including setting a quit date. The proactive telephone peer support was provided by a woman ex-smoker. Ongoing calls typically occurred on a weekly basis, but more frequently around a quit date, and less frequently as smoking changes stabilised. On average, the calls lasted 10 minutes, and the woman could elect to stop receiving the calls at any time | Other HCP | Combination | NR |
| Patten (2020) | Peer counselling with social marketing campaign | Counselling | Multi-level | Access to usual care. Locally adapted, community-wide social marketing campaign and, for enrolled pregnant women, individual peer phone counselling. Both components were delivered by 5 local AN “Native Sisters”. Campaign involved mailing of DVDs to residents and Facebook posts around harms of smoking to mother and baby. Peer counselling; up to 3 calls during pregnancy (weeks 1,2 & 4 post enrolment) and 3 calls in the postpartum period (weeks 2, 4 & 6 after childbirth). Session content included evidence-based techniques for being tobacco-free, based on the 5 As. Evidence-based techniques for quitting tobacco/remaining tobacco-free provided by the NSs were providing support, problem solving, and reinforcement. | Other HCP | Combination | From enrolment to 6 weeks after childbirth |
| Robling (2015) | Family Nurse Partnership (FNP) programme | Counselling | Single | Usually provided care, plus FNP. FNP involves up to 64 structured home visits by specially recruited and trained family nurses. Developed in the USA for first-time pregnant women, it was adapted under licence for delivery in England from early pregnancy until children were 2 years old. FNP aims to affect risks and protective factors within prenatal health-related behaviours, sensitive and competent caregiving, and early parental life course. Core specialist training for nurses includes motivational interviewing and the adoption of a guiding autonomy-supportive communication style with clients. | Nurse | In person | NR |
| Bradizza (2017) | Cognitive-Behavioural Smoking Cessation Treatment and Emotional Regulation Treatment | Counselling | Single | Programme comprising three major components: (1) coping skills for managing negative emotions with the goal of developing emotional awareness and providing skills to modulate negative affect and tolerate distress, (2) guided imagery (imaginal exposure) to elicit negative emotions consistently associated with smoking and promote emotional processing of these triggers, and (3) mindfulness meditation skills including mindful breathing, mindfulness in daily activities and “urge surfing”. | Unclear | Unclear | 8 sessions delivered before childbirth |
| Dornelas (2006) | Smoking cessation counselling | Counselling | Single | Access to usual care, plus 90-min psychotherapy session, followed by bi-monthly prenatal telephone calls from the therapist during pregnancy, and monthly telephone calls after delivery. The goal was to use techniques of brief psychotherapy to assist women in recognizing the link between psychological distress and inability to quit smoking when pregnant. The conceptual foundation of the model assumed that behaviour change is more likely to occur when the patient: (1) experiences affective arousal during the counselling session, (2) experiences a high degree of interpersonal engagement with the therapist, and (3) recognizes that smoking can be a way of coping with emotional distress. | Counsellor | Combination | Throughout pregnancy and postnatal period |
| Secker-Walker (1997) | Videotape advice | Counselling | Single | Four lower-income women smokers, ages 25-29 years, were recruited to be videotaped going through the process of quitting smoking. The videotape follows each woman as she describes why she decided to quit, what her quitting plans were, how she felt at the end of her quit day, and how things were going during the subsequent two to three months. Topics discussed included; how they were coping with negative feelings, urges to smoke, and weight gain, and also the ways they were getting support from their families and friends. Usual care also received | No facilitator | Self-support | NR |
| Hennrikus (2010) | Peer support | Social support | Multiple | Single counselling session for all subjects was designed to increase motivation to quit and provide information about community smoking cessation resources. Plus, supporter sessions to develop strategies to help the subject quit smoking. The Counsellor provided regular calls to the supporter to identify activities to improve efforts to quit. Intervention subjects and supporters received materials to create a pregnancy scrapbook that included pages related to the smoking cessation tasks. The scrapbook was intended to facilitate interaction between dyads. | Peer supporter | Unclear | NR |
| Tuten (2012) | Financial incentives | Incentives | Single | Earn vouchers contingent on smoking reduction or abstinence for a period of 12 weeks or until delivery. Smoking targets were minimal during the initial weeks of intervention, and increased gradually to ensure adequate learning and reinforcement. $7.50 voucher for the first smoking reduction target, and the value of the voucher increased by $1/day for each consecutive target met throughout the 12-week incentive period to a maximum of $41.50. | Unclear | In person | 12 weeks or until delivery |
| Brandon (2012) | Forever Free tailored smoking cessation booklets | Health education | Single | The first 4 booklets (Overview; Smoking Urges; Smoking and Health; A Time of Change) were mailed over equal intervals between the date of a participant’s enrolment in the study and her expected due date. The next 5 booklets (What If You Have a Cigarette? Smoking, Stress and Mood; Lifestyle Balance; Smoking and Weight; Life Without Cigarettes) were mailed at 1, 2, 3, 4, 6, and 8 months postpartum. Partner Support was mailed with the first booklet, including instructions to deliver it to the participant’s primary partner. | No facilitator | Self-support | To 8 months postpartum |
| Hajek (2001) | Midwife advice | Health education | Multiple | Midwife advice to stop smoking including interpretation of the CO reading, written materials regarding harms of smoking, advice and tips of abstinence, avoiding relapse and addressing myths around association of smoking reducing stress. Quiz at the end of each section of the book. Midwife checked answers and addressed any incorrect responses. Commitment to stop smoking co-signed by midwife and partner. Opportunity to be paired with another quitter for peer support. Reinforcement of stop smoking messages at future midwifery appointments | Midwife | In person | NR |
| Secker-Walker (1998) 1a | Structured advice and individual counselling | Counselling | Single | Protocol for advice given by Physician, including; acknowledgement of smoking, interpretation of CO result, advice on harms, eliciting commitment to quit and advising of support from Counsellor. Individual counselling from a trained Nurse provided at first, second, third and fifth prenatal visits, and again at 36th week of pregnancy | Nurse | Unclear | To 36th week of pregnancy |
| Secker-Walker (1998) 1b | Structured advice and individual counselling | Counselling | Single | structured advice from their physician and referral to individual relapse prevention counselling at the first, second, third, and fifth prenatal visits, and again at the 36-week visit. At the first visit, the structured advice was similar to the usual care advice, but in addition the physician asked how the woman felt about staying quit, offered help, and then referred the woman to individual relapse prevention counselling provided on site by a trained nurse. During the individual counselling, the woman received praise for not smoking and then assistance with ways to cope with and resist urges and temptations to smoke. At the second, third, and fifth visit, the same protocol was followed for women who continued to report not smoking with referral to the nurse counsellor at each of these visits. Where women had started smoking again, they were encouraged to set a new quit date and provided with strategies and advice to help stay quit | Nurse | Unclear | To 36th week of pregnancy |
| Reitzel (2010) | Smoking cessation counselling (MAPS) | Counselling | Single | In addition to usual care, six telephone-based counselling sessions (Weeks 34 and 36 prepartum and Weeks 2, 4, 7, and 16 postpartum) | Counsellor | Telephone | From week 34 of pregnancy to 16 weeks postpartum |
| Ondersma (2011) | Peer and professional cessation advice plus multi-media cessation plan (CD5As) | Counselling | Single | Video of professional and those with lived experience about risks of smoking in pregnancy and testimonials. Advice to quit from the Obstetrician, whose advice to quit using gain-framed messages. Following the tailored video, the software completed the remaining 5As elements using a combination of narrated graphics, feedback, education, and interactive questioning using branching logic and reflective responses. the software provided assistance in developing a specific plan and provided a menu of options involving the three most readily available approaches: self-help, calling the local quit line (1-800-QUITNOW), and talking with the doctor or nurse | No facilitator | Digital | NR |
| Hebel (1985) | NR | Unclear | Single | received immediate assistance with smoking cessation. Contacts were maintained with the treatment group throughout their pregnancy to help the women to stop smoking. | Unclear | Unclear | NR |
| Albrecht (1998) | Teen Freshstart programme | Counselling | Single | CBT group model designed for adolescents. he goals of the TFS program are to increase the smoker’s awareness of her own smoking patterns, identify triggers to smoke, and provide accurate information on the effects of smoking on health. The investigators modified the TFS model to include additional information related to the effects of smoking on pregnancy and the foetus, as well as body image changes and overall health. Social activities, immediate rewards (gifts and refreshments), and adult modelling were incorporated into the program to enhance subject attendance and participation. | Unclear | In person | Eight sessions in the prenatal period |
| Stotts (2004) | Motivational interviewing | Counselling | Single | MI intervention over the course of 8 weeks: (1) one face-to-face MI session; (2) three MI-based telephone counselling calls; and (3) one personalized feedback letter providing assessment results. | Counsellor | Combination | Eight sessions during prenatal period |
| El-Mohandes (2011) | Smoking cessation intervention | Counselling | Single | Consistent with the SCRIPT procedures and the Counselling and Behavioural Interventions Work Group of the United States Preventive Services Task Force recommendations, a five-step behavioural counselling approach. The intervention was tailored to the woman’s stage of change. Women were encouraged to avoid triggers and to use alternative coping and behavioural change strategies. Intervention included strategies for reducing exposure to environmental tobacco smoke | Unclear | Unclear | 8 sessions in the prenatal period and 2 in the postpartum period |
| Rigotti (2006) | Smoking cessation counselling | Counselling | Single | In addition to usual care a series of telephone calls accompanied by additional mailed written materials. Each subject had a dedicated counsellor who offered up to 90 minutes of counselling during pregnancy and up to 15 minutes of counselling over 2 months postpartum. The counsellor tailored the schedule of calls to the subject’s needs. Counselling targeted pregnancy-related issues and was individually tailored to each subject’s readiness to quit and interest in other pregnancy and health-related topics. During the initial call, the Counsellor and pregnant woman developed a plan for cessation or reduction. After the initial call, subjects were mailed a personalized worksheet. After each call, they received a summary letter and targeted written materials. | Counsellor | Telephone | Until 2 months postpartum |
| Cinciripini (2010) | Cognitive Behavioural Analysis System of Psychotherapy | Counselling | Single | Treatment strategy using a social problem-solving exercise called Situational Analysis, which is a technique used to create awareness of the contingent relationship between participants' behaviour and outcomes in stressful interpersonal situations. participants were first asked to identify a recent, distressing interpersonal or smoking-related situation. During elicitation, participants were asked to describe the event, their interpretations of what occurred, their behaviours during the event, the actual outcome of the event, their desired outcome, and whether the desired outcome was achieved. In the remediation phase, the therapist worked with participants to modify their interpretation of the event, their behaviour and/or desired outcome, such that the likelihood of achieving the desired outcome would be increased. In the generalization phase, participants learned to apply these new skills or understandings to other situations. | Counsellor | Telephone | 10 prenatal individual counselling visits |

*NR = not reported, HCP = healthcare professional*

# **Appendix 2 table 3 - sample characteristics of included studies**

| **Author (year)** | **Target population** | **Participant inclusion criteria** | **ITT sample** | **Age (mean, SD)** | **Weeks’ gestation at enrolment (mean, SD)** | **Cigarettes per day smoked (mean, SD)** | **Ethnicity (%)** | **Socioeconomic status (%)** |
| --- | --- | --- | --- | --- | --- | --- | --- | --- |
| Abroms (2017) | Smokers | ≥14 years, currently pregnant with due date >8 weeks from recruitment text being sent, had smoked at least one puff of a cigarette in the past 2 weeks and had personal cell phone | 497 | 26.31 (5.85) | 17.8 (7.92) | 7.36 (6.09) | White: Non-Hispanic, Latino or Spanish 63.18%, Black/African American: Non-Hispanic, Latino or Spanish 23.54%, Other: Non-Hispanic, Latino or Spanish 5.03%, Hispanic, Latino or Spanish 8.05% | Medicaid/medicare 80.05%. Household income <$15,000 54.92%, $15,000 - $30,000 29.92%, £30,000 - $47,099 9.26%, > $47,100 3.82% |
| Ershoff (1999) | Smokers | Smokers including those who had cut back since becoming pregnant, and those reporting smoking from time to time | 332 | 29.4 (NR) | NR | 17 (NR) | White: 60% | Some college education: 50% |
| Forinash (2018) | Smokers | Active smokers, at least 18 years of age with a confirmed pregnancy, with obstetric care provided at the MFCC, English speaking, in the preparation stage of change using the transtheoretical model of change, willing to set a quit date before 35 weeks’ gestation, had the ability to receive text messages and be willing to pay for any related fees. | 49 | NR | NR | NR | Black (76.7%), White (23.3%) | Public insurance: 100% |
| Windsor (1993) | Smokers | NR | 814 | 24.6 (NR) | NR | NR | Black: 52% | Mean education: 12.4 years |
| Alaniz (2019) | Smokers & spontaneous quitters | Pregnant women enrolling in First Breath in any of the target counties, including, current smokers and those who had been a daily smoker sometime in the last 6 months | 185 | 28.5 (NR) | 27.1 (NR) | NR | Black/African American = 54.4%, White = 31.3%, Bi- or multi- = 8.8%, Other 5.5% | Household income <$10,000 = 54.2%, Not employed = 59% |
| Baker (2018) | Smokers & spontaneous quitters | Female, pregnant, not involved in another stop smoking research study. Willingness to quit or cut down on smoking in the next 30 days or, if already quit, desire to stay quit after the birth. Daily smoker (at least one cigarette each day for at least one week) at some time point within the last 6 months, health insurance coverage by the Wisconsin Medicaid program, enrolled in a participating HMO. | 1014 | 26 (NR) | 14 (NR) | 10 (NR) | Racial minority = 50% | High school education = majority |
| Bullock (2008) | Smokers | Women attending 21 rural Women Infant and Children Nutritional Supplement (WIC) clinics in a Midwest state who reported smoking at least 1 cigarette per day, spoke English, and were 18 years or older and less than 24 weeks gestation. | 530 | 22 (NR) | 13.5 (NR) | NR | White = 91%, African American = 3.5%, Hispanic = 1.7%, Asian = 0.3%, Native American = 1.4%, Other = 2.5% | High school diploma or GED = 63% |
| Coleman-Cowgear (2018) | Smokers | First or second trimester of pregnancy, aged 18 or older, and self-reported tobacco use in the past 90 days | 128 | 26 (NR) | NR | 8.6 (NR) | African American = 80.5%, White = 15.6%, Other = 3.9% | Unemployed/not working = 78%. |
| Eades (2012) | Smokers & spontaneous quitters | Aboriginal or Torres Strait Islanders; attending their first antenatal appointment at one of the Aboriginal community-controlled health services at or before 20 weeks’ gestation; aged 16 years or older, self-reported current smokers or recent quitters (quitting when they knew they were pregnant); and residents of the local area. | 263 | NR | 12 (4) | NR | Aboriginal or Torres Islanders = 100% | NR |
| Gielen (1997) | Smokers | Any woman who indicated that she had 'smoked a cigarette—even one puff—in the past 7 days', <28 weeks pregnant, African-American or White | 391 | 23 (NR) | 16 (NR) | 8 (NR) | Majority African American <80% | 50% less than high school educated |
| Glover (2015) | Smokers | Women aged ≥16 years, self-identified as Māori, resided in the Auckland region, were 2–30 weeks pregnant, were daily smokers, able to provide written consent and have access to a telephone or email. | 24 | 25 (2.25) | NR | 9 (NR) | 100% Māori | Majority secondary (years 11-12) or trade/vocation educated |
| Kendrick (1995) | Smokers | Every woman who reported having smoked even a puff of a cigarette within 7 days before screening or within 7 days before she thought she was pregnant was considered a smoker and was asked for consent to collect questionnaire data and urine specimens. | 1120 | NR | 19 (NR) | NR | <71% White in each state. | Receiving WIC support ranged from 14% - 43% across states. Income <$500 per month ranged from 27% - 40% across states. |
| Klerman (2011) | Smokers | (1)African American, (2) eligible for Medicaid,(3) less than 26 weeks’ gestation, (4) at least 16years old, and (5) score of 10 or higher on a risk assessment scale. The scale was based on multiple analyses of a computerized database that included all Medicaid-eligible pregnancies in the area during 1993 and 1994 and a prospective study of high-risk pregnancies | 48 | 22.5 (NR) | 11 (NR) | NR | 100% African American | 100% Medicaid eligible |
| Lee (2015) | Smokers | (1) pregnant (in their first trimester), (2) had smoked one puff of a cigarette in the 30 days prior to recruitment, (3) 18 years or older, (4) reachable by a telephone | 277 | 27 (NR) | NR | 7.6 (7.4) | African-American, 56%; Hispanic, 12 % | Income 50 % <$15,000, education 65% <high school |
| Lowe (1998) 1a | Smokers | In the first trimester of pregnancy, had no current complications and were not planning to give the child up for adoption at birth | 217 | NR | NR | NR | NR | NR |
| Lowe (1998) 1b | Smokers | In the first trimester of pregnancy, had no current complications and were not planning to give the child up for adoption at birth | 78 | NR | NR | NR | NR | NR |
| Mejdoubi (2014) | Smokers | Phase 1 - (1) maximum age of 25 years, (2) low educational level (primary school or prevocational secondary school), (3) maximum 28 weeks of gestation, (4) no previous live births and (5) understanding of the Dutch language. Phase 2 - (1) identification of at least one additional risk factor. | 460 | 19.4 (NR) | 19.8 | 07-Aug | 49% Dutch | 29% employed |
| Patten (2019) | Smokers | (1) AN woman eligible for care at SCF PCC, (2) aged >18 years, (3) willing to provide written informed consent, (4) currently pregnant (≤28 weeks’ gestation with a singleton pregnancy), (5) resided in Anchorage, (6) planned to deliver at the Alaska Native Medical Centre, (7) current smoker (any cigarettes smoked during the past seven days), and (8) willing to enrol into the SCF clinical “Quit Tobacco Program”. Participants could use other forms of tobacco if cigarettes were the primary tobacco used. | 30 | 27 (5) | 14-15 (6.5) | NR | 100% Alaskan Native | Majority some college education |
| Patten (2010) | Smokers | (a) ≥18 years, (b) ≤24 weeks gestation, (c) self-reported smoking or Iqmik/ST use in the last 7 days, (d) planning to quit in the next 30 days, (e) access to a telephone and VCR/DVD player, and (f) willing to participate in all study procedures. | 33 | 25 (5) | 13 (5) | NR | Yupik ethnicity 100% | Majority less than high school or high school educated |
| Pbert (2004) | Smokers & spontaneous quitters | Smokers or had spontaneously quit after learning of their pregnancy (and had not smoked for at least 7 days prior to baseline assessment) | 550 | 25.7 (6) | NR | 16 (11.50) | 53% White, 20% Black, 19% Hispanic, 8% Other | 62% Medicaid, 70% not in paid work |
| Polanska (2004) | Smokers & spontaneous quitters | Admitted smoking at first prenatal appointment or having spontaneously quit upon learning they were pregnant | 442 | 25.5 (6.2) | 19.6 (8) | 7.5 (NR) | NR | Most primary or vocational educated and most not in employment |
| Polanska (2005) | Smokers & spontaneous quitters | Admitted smoking at first prenatal appointment or having spontaneously quit upon learning they were pregnant | 182 | NR | NR | NR | NR | NR |
| Price (1991) | Smokers | NR | 193 | 22.6 (5.6) | NR | NR | 70% White | 87% not graduated from High School |
| Ruger (2008) | Smokers & spontaneous quitters | 1) being pregnant for less than 28 weeks and receiving prenatal care at a participating site; 2) being a current smoker (smoking cessation) or having been a smoker within 3 months of baseline (relapse prevention); 3) not receiving inpatient drug treatment; and 4) speaking English or Spanish | 310 | 26 | NR | NR | 67% White, 17% Black, 9.6% Hispanic, 1% American Indian, Aleut or Eskimo, 0.65% Asian/Pacific Islander, 13.6% Other | 32% <high school, 41% completed high school, 26% postsecondary |
| Tappin (2022) | Smokers | self-reported smokers (at least one cigarette in the past seven days), 16 years or older, less than 24 completed weeks of gestation, and English speakers for verbal telephone consent. | 472 | 27.9 (5.8) | 11.3 (3.3) | 10 (NR) | 98.5% White | 43% living in most deprived quintile (IMD) |
| Windsor (2011) | Smokers | NR | 1093 | 22.9 (NR) | 9.5 | 10.6 (NR) | 16.3% Black | 100% Medicaid eligible |
| Windsor (2000) | Smokers | NR | 265 | 23 (NR) | 10.4 | 10 (NR) | 16% Black | 100% Medicaid eligible |
| Stretcher (2000) | Smokers & spontaneous quitters | Those who reported having smoked at least 100 cigarettes in their lifetime and who were either still smoking or had quit since becoming pregnant. | 173 | 26.2 (5.6) | 17 (8) | 19.5 (10.3) | 84% White | Mean education: 12.5 years (SD 2) |
| Donatelle (2000) | Smokers | >15 years, self-reported smoker ("even a puff in the last seven days"); English speaker/reader, WIC eligible; and 28 weeks’ gestation or less | 207 | 23.75 (5.75) | 16.5 (7) | NR | 11% not White, 8% Latina or Hispanic | Mean education: 11.7 years (SD 1.85); 88% with household income < $20000 |
| Langford (1983) | Smokers & spontaneous quitters | Daily smoker at any time during pregnancy or three months prior to pregnancy | 116 | 25 (5) | NR | NR | NR | Higher level of education than women in Canada generally |
| Mayer (1990) | Smokers | NR | 219 | 22.7 (NR) | 26 (NR) | 19.9 (NR) | 75% White, 20.8% Black, 4.2% Other | 76.5% Medicaid |
| Panjari (1999) | Smokers | Current smoker, gestation less than 20 weeks, singleton pregnancy, the ability to speak and read English, and no drug dependency that would prompt referral out of the main antenatal clinic to the Chemical Dependency Unit. | 732 | 26 (NR) | 12 | 21 (NR) | NR | NR |
| Malchodi (2003) | Smokers | 1) current smoker (smokes at least one cigarette per day the week before learning of pregnancy), 2) documented pregnancy with intention to carry to term, 3) less than 20 weeks’ gestation, 4) speaks either English or Spanish, and 5) is 18 years or older. | 142 | 25.5 (6) | NR | 12.25 (8.3) | 63% Hispanic, 12.5% Black, 23.5% White, 1%. Other | 61.5% unemployed, majority 9-11 grade education |
| Burling (1991) | Smokers | Smokers identified by either self-report or breath CO content (9 ppm or greater) | 139 | NR | NR | NR | 61.7% Caucasian, 40.4% Black, 8.3% Asian | NR |
| Lilley (1986) | Smokers | Anyone currently smoking one cigarette or more per day | 145 | 23.85 (NR) | NR | NR | NR | NR |
| Soloman (2000) | Smokers | Pregnant women who reported smoking at least one cigarette in the past week | 151 | 23.5 (6.15) | 11.6 (5.25) | 21.4 (10.7) | 95% White (race) 98.7% non-Hispanic ethnicity | Mean education = 11.6 years (SD 2) 76% Medicaid insurance |
| Patten (2020) | Smokers & spontaneous quitters | (1) Alaskan Native woman; (2) aged ≥18 years; (3) currently pregnant and at ≤36 weeks of gestation at the time of screening; and (4) had access to a working telephone | 352 | 28.5 (5) | 26.8 (9.8) | NR | 100% Alaskan Native | 44% working/employed, 25% less than high school education, 60% high school education, 16% beyond high school education |
| Robling (2015) | Smokers | nulliparous, aged 19 years or younger, living within the catchment area of a local FNP team, of less than 25 weeks’ gestation, and able to provide consent and speak English. Women expecting multiple births and those with a previous pregnancy ending in miscarriage, stillbirth, or termination were eligible. | 1092 | 17.9 (NR) | 16 (NR) | NR | 88% White, 5.5% Mixed, 1.5% Asian, 4.5% Black, <1% Other | 79% no paid job, 48% NEET status |
| Bradizza (2017) | Smokers | (1) age 18 years or older, (2) singleton pregnancies, (3) smoked, on average, at least 1 cigarette per day over the prior week, (4) less than 24 weeks gestation, (5) negative affect smoker as defined by a mean of 5.6 or greater on the negative affect reduction scale of the Brief Smoking Consequences Questionnaire-Adult, (6) able to provide a collateral contact, defined as a person who is familiar with their smoking, (7) no more than 0.50 ounces of ethanol (1 drink per day) and no incidence of binge drinking during pregnancy (≥4 drinks per occasion). | 70 | 24.8 (4.6) | 15.3 (5) | 7.5 (11.7) | 42.9% African American, 29.8% Caucasian, 15.85% Hispanic, 5.75% Other | Household income <$10,000 per year 69%, $10,000 - $20,000 21%, >$20,000 10% |
| Dornelas (2006) | Smokers | women ≥ 18 years old and ≤30 weeks’ gestation who were current smokers | 105 | 26.1 (5.8) | 24 (5.89) | 10 (8.90) | 66% Hispanic, 17% Caucasian, 11% African American and 6% multi-racial or Other ethnic category, | 61% unemployed, 54% had less than a full high school education, 49% household income of ≤$15,000/year. |
| Secker-Walker (1997) | Smokers | Women smoking in average of one or more cigarettes a day at their first prenatal visit | 49 | 23 (5.5) | NR | 22.6 (7.4) | 99% White | 33% <high school education |
| Hennrikus (2010) | Smokers | In the first or second trimester, a current smoker, and at least 18 years old. | 82 | NR | NR | 5 (NR) | 67% percent of the subjects were from racial minorities or were Hispanic, | 65% <high school education |
| Tuten (2012) | Smokers | NR | 102 | 30.8 (6) | 16.6 (6.9) | 18 (8.6) | 65% Caucasian, 35% African American | 94.7% Unemployed |
| Brandon (2012) | Smokers & spontaneous quitters | > 18 years old; able to speak and read English; currently in months 4 through 8 of pregnancy; previously smoked at least 10 cigarettes per day for at least 1 year before pregnancy; quit smoking either in anticipation of, or during pregnancy; and had abstained for the past week. | 504 | 25.8 (5.5) | NR | 15.2 (6.6) | 92% White, 4.5% Black, 5.8% Hispanic, 3.3% Other | 41.5% employed, 9% <high school diploma |
| Hajek (2001) | Smokers & spontaneous quitters | Current smokers or stopped smoking up to 3 months previously (recent ex-smokers), and provided informed consent. | 1120 | 27.6 (5.8) | NR | 10.8 (6.7) | NR | 20% no educational attainment |
| Secker-Walker (1998) 1a | Smokers | Women smoking 1 or more cigarettes at their first prenatal visit | 276 | 22.5 (5.1) | 14.9 (7.2) | 25.6 (11.6) | NR | 72.5% on Medicaid |
| Secker-Walker (1998) 1b | Smokers & spontaneous quitters | Women who had smoked before pregnancy but reported quitting smoking before their first prenatal visit | 92 | 21.4 (4.3) | 16.25 (8) | 13.8 (8.8) | NR | 66.6% on Medicaid |
| Reitzel (2010) | Smokers & spontaneous quitters | English-speaking pregnant women aged 18 years or older who stopped smoking either during their pregnancy or within 2 months prior to becoming pregnant. | 251 | 24.6 (5.3) | 32 | 10.2 (7.6) | 32% Black, 30% Latino, and 36% White | 55% <$30,000/year in annual household income |
| Ondersma (2011) | Smokers | age 18 years or older, being no further than 27 weeks into gestation, and reporting smoking in the past week (while pregnant) | 104 | 27.9 (6.4) | 20 (NR) | 8 (8.2) | 81.8% Black, 1 participant Hispanic | NR |
| Hebel (1985) | Smokers | Pregnant women who were smoking at least 10 cigarettes per day at the beginning of pregnancy and who had not passed their 18th week of gestation |  | 24.9 (4.8) | 15 (NR) | 20.8 (11.2) | 60% White, 40% Black | Mean years of education - 12.3 (SD 2.1) |
| Albrecht (1998) | Smokers | 12-20 years of age, 4-28 weeks’ gestation, single marital status, reported smoking at least 1 cigarette per day, no previous live births, and ability to read and write English. | 84 | NR | NR | NR | 63% African American heritage, 37% American-European heritage | NR |
| Stotts (2004) | Smokers | At least 16 years of age, fluent in English, less than 28 weeks’ gestation, and reported smoking in the past 7 days | 54 | NR | NR | NR | NR | NR |
| El-Mohandes (2011) | Smokers & spontaneous quitters | 18 years or older, English-speaking, less than 29 weeks’ gestation and Washington, DC residents, having smoked a puff of a cigarette or more in the 6 months preceding pregnancy | 384 | 26.8 (6.3) | NR | NR | 100% African-American | 89% Medicaid |
| Rigotti (2006) | Smokers | Had smoked at least 1 cigarette in the past 7 days, were 18 years of age or older, at 26 weeks or less of gestation, willing to consider altering their smoking during the pregnancy, reachable by telephone, English-speaking, and expected to live in New England for the next year. | 421 | 28.5 (6.25) | 12.7 (4.6) | 20.8 (8.7) | 88% non-Hispanic, White | Mean 13 years in education (SD 2), 73% Private Health Insurance, 89% employed in the last year |
| Cinciripini (2010) | Smokers | ≥ 16 years of age, ≤ 32 weeks pregnant, have smoked at least a puff or more during the past 7 days, have a telephone, and express a willingness to quit smoking during the study (i.e., women with a goal of reducing cigarette consumption only were not eligible) | 257 | 25 | NR | 10 (NR) | 54% African American, 33.5% White, 9.3% Hispanic, 3.1% Other | 31.9% Less than high school educated, 35% High school/GED. 34% < $10,000 family income, 14% $10,000 – $19,999, 4% $20,000 – $29,999, 20.6% >$30,000 |

*NR = not reported, WIC = women and infant clinic, NEET = not in employment, education or training, GED= General Educational Development program, AN= Alaskan Native, FNP= Family Nurse Partnership, IMD= Index of Multiple Deprivation*

# **Appendix 2 table 4 - study level effects of included studies**

| **Author (year)** | **Intervention name (I)** | **Control name (C)** | **Outcome description** | **Measure** | **No. (I)** | **Prop. (I)** | **No. (C)** | **Prop. (C)** | **OR** | **LCI** | **UCI** | **P value** |
| --- | --- | --- | --- | --- | --- | --- | --- | --- | --- | --- | --- | --- |
| ***Prenatal smoking cessation*** | | | | | | | | | | | | |
| Bradizza (2017) | CBT with emotional regulation treatment | CBT + HLS | Biochemically validated 7-day PPA 2 months after quit date | Urine cotinine | 5 | 23.00% | 0 | 0.00% | 13.51 | 0.7 | 261.6 | 0.085 |
| Bradizza (2017) | CBT with emotional regulation treatment | CBT + HLS | Biochemically validated 7-day PPA 4 months after quit date | Urine cotinine | 3 | 18.00% | 1 | 5.00% | 2.98 | 0.39 | 22.72 | NR |
| Bradizza (2017) | CBT with emotional regulation treatment | CBT + HLS | Biochemically validated 7 day PPA 4 months after quit date | Urine cotinine | 3 | 18.00% | 1 | 5.00% | 3.86 | 0.36 | 41.2 | 0.264 |
| Cinciripini (2010) | CBASP | HW | Biochemically verified 7 day PPA at the end of treatment | CO reading | 58 | 45.30% | 51 | 39.20% | 1.2 | 0.7 | 2 | NR |
| Cinciripini (2010) | CBASP | HW | Self-reported contiuous abstinence 3 months post treatment | Self-report | 30 | 23.40% | 27 | 21.00% | 1.2 | 0.6 | 2.1 | NR |
| Cinciripini (2010) | CBASP | HW | Self-reported 7 day PPA 3 months post treatment | Self-report | 47 | 36.70% | 40 | 31.00% | 1.3 | 0.8 | 2.2 | NR |
| Cinciripini (2010) | CBASP | HW | Self-reported prolonged abstinence 3 months post treatment | Self-report | 40 | 31.30% | 35 | 27.10% | 1.2 | 0.7 | 2.1 | NR |
| Dornelas (2006) | Smoking cessation counselling | Usual care | Biochemically validated 7 day PPA at end of pregnancy | CO reading | 15 | 28.30% | 5 | 9.60% | 3.71 | 1.24 | 11.13 | 0.0193 |
| Forinash (2018) | Smoking cessation programme with text messaging | SOC smoking cessation programme | 2-week cessation verified by eCO | CO reading | 8 | 34.78% | 5 | 19.23% | 2.24 | 0.61 | 8.21 | 0.2237 |
| Forinash (2018) | Smoking cessation programme with text messaging | SOC smoking cessation programme | 2-week cessation verified by eCO | CO reading | 5 | 21.74% | 2 | 7.69% | 3.33 | 0.58 | 19.18 | 0.1775 |
| Kendrick (1995) | Counselling and cessation information | Usual prenatal care | Biochemically verified smoking abstinence in the 8th month of pregnancy (aggregated 3 state) | Urine cotinine | 54 | 6.10% | 69 | 5.90% | 1 | 0.69 | 1.6 |  |
| Kendrick (1995) | Counselling and cessation information | Usual prenatal care | Biochemically verified smoking abstinence in the 8th month of pregnancy (Colorado) | Urine cotinine | 19 | 8.20% | 26 | 9.20% | 1 | 0.31 | 3.3 | NR |
| Kendrick (1995) | Counselling and cessation information | Usual prenatal care | Biochemically verified smoking abstinence in the 8th month of pregnancy (Maryland) | Urine cotinine | 22 | 7.20% | 28 | 5.10% | 1.2 | 0.01 | 86 | NR |
| Kendrick (1995) | Counselling and cessation information | Usual prenatal care | Biochemically verified smoking abstinence in the 8th month of pregnancy (Missouri) | Urine cotinine | 13 | 6.10% | 15 | 5.90% | 1 | 0.69 | 1.6 | NR |
| Kendrick (1995) | Counselling and cessation information | Usual prenatal care | Self-reported smoking abstinence in the 8th month of pregnancy (aggregated 3 state) | Self-report | 190 | 13.00% | 168 | 9.50% | 1.4 | 1.2 | 1.9 | NR |
| Kendrick (1995) | Counselling and cessation information | Usual prenatal care | Self-reported smoking abstinence in the 8th month of pregnancy (Colorado) | Self-report | 65 | 13.90% | 48 | 10.30% | 1.6 | 1 | 2.7 | NR |
| Kendrick (1995) | Counselling and cessation information | Usual prenatal care | Self-reported smoking abstinence in the 8th month of pregnancy (Maryland) | Self-report | 49 | 11.80% | 73 | 9.80% | 1.1 | 0.18 | 7.3 | NR |
| Kendrick (1995) | Counselling and cessation information | Usual prenatal care | Self-reported smoking abstinence in the 8th month of pregnancy (Missouri) | Self-report | 76 | 13.00% | 47 | 8.40% | 1.6 | 0.89 | 3.1 | NR |
| Lee (2015) | Cognitive behavioural counselling | Best practice care | Biochemically 7-day PPA pre-partum | Saliva cotinine | 21 | 15.00% | 16 | 11.70% | 1.32 | 0.66 | 2.7 | 0.43 |
| Lowe (1998) 1a | Smoking cessation counselling | Brief advice | Biochemically validated 7-day PPA at 20 weeks’ gestation | Urine cotinine | 3 | 2.80% | 3 | 2.10% | 1.05 | 0.21 | 5.31 | 0.9543 |
| Mayer (1990) A/C | Behaviour change counselling | Usual care | Smoking abstinence at the 9th month of pregnancy | Self-report | 8 | 11.11% | 2 | 2.60% | 4.69 | 0.96 | 22.87 | 0.0561 |
| Mayer (1990) B/C | Risk information | Usual care | Smoking abstinence at the 9th month of pregnancy | Self-report | 5 | 7.14% | 2 | 2.60% | 2.88 | 0.54 | 15.37 | 0.2146 |
| Mejdoubi (2014) | Voorzorg Nurse Programme | V-MIS | Smoking cessation at 32 weeks pregnancy | Self-report | 162 | 68.35% | 154 | 69.06% | 0.97 | 0.65 | 1.44 | 0.8708 |
| Ondersma (2011) A/D | CD5As | Usual care | Biochemically validated abstinence 10 weeks after randomisation | Urine cotinine | 10 | 43.50% | 4 | 17.40% | 10.1 | 1.4 | 75 | < .05 |
| Ondersma (2011) A/D | CD5As | Usual care | Biochemically validated 7-day PPA 10 weeks after randomisation | CO reading | 7 | 30.40% | 2 | 8.70% | 5.7 | 0.9 | 34.3 | > .05 |
| Ondersma (2011) A/D | CD5As | Usual care | Self-reported continuous 30-day abstinence in pregnancy | Self-report | 6 | 26.10% | 1 | 4.30% | 14.2 | 1.2 | 172.3 | < .05 |
| Ondersma (2011) C/D | CD5As + CM-LITE | Usual care | Biochemically validated abstience 10 weeks after randomisation | Urine cotinine | 4 | 15.40% | 4 | 17.40% | 0.7 | 0.1 | 4 | > .05 |
| Ondersma (2011) C/D | CD5As + CM-LITE | Usual care | Biochemically validated 7-day PPA 10 weeks after randomisation | CO reading | 5 | 19.20% | 2 | 8.70% | 2.8 | 0.5 | 16.9 | > .05 |
| Ondersma (2011) C/D | CD5As + CM-LITE | Usual care | Self-reported continuous 30 day abstinence in pregnancy | Self-report | 5 | 19.20% | 1 | 4.30% | 7 | 0.6 | 81.2 | > .05 |
| Panjari (1999) | Personalised care | Usual care | Biochemically confirmed smoking abstinence in late pregnancy | Urine cotinine | 33 | 9.73% | 31 | 7.89% | 1.26 | 0.75 | 2.1 | 0.3786 |
| Patten (2010) | Multi-media education pack and counselling | Usual care | Self-reported 7-day PPA at end of pregnancy | Self-report | 1 | 6.00% | 1 | 6.00% | 1.07 | 0.06 | 18.6 | 0.9647 |
| Patten (2010) | Multi-media education pack and counselling | Usual care | Biochemically validated 7-day PPA at end of pregnancy | Saliva cotinine | 0 | 0.00% | 1 | 6.00% | 0.33 | 0.01 | 8.79 | 0.5106 |
| Patten (2020) | Peer counselling with social marketing campaign | Usual care | Self-reported 7-day PPA at time of delivery (all participants at baseline) | Self-report | 66 | 35.11% | 53 | 32.32% | 1.13 | 0.73 | 1.77 | 0.5811 |
| Patten (2020) | Peer counselling with social marketing campaign | Usual care | Self-reported 7-day PPA at time of delivery (tobacco users only at baseline) | Self-report | 22 | 18.33% | 16 | 14.04% | 1.38 | 0.68 | 2.78 | 0.3741 |
| Patten (2020) | Peer counselling with social marketing campaign | Usual care | Self-reported 7-day PPA at time of delivery (non-tobacco users only at baseline) | Self-report | 44 | 64.71% | 37 | 74.00% | 0.64 | 0.29 | 1.44 | 0.2837 |
| Polanska (2004) | Midwife delivered smoking cessation programme | Usual care | Self-reported smoking abstinence in pregnancy (smokers only at baseline only) | Self-report | NR | 44.30% | NR | 16.70% | 6 | 4.6 | 7.7 | NR |
| Polanska (2004) | Midwife delivered smoking cessation programme | Usual care | Self-reported smoking abstinence in pregnancy (all participants) | Self-report | NR | 48.30% | NR | 33.70% | 2.5 | 1.8 | 3.7 | NR |
| Robling (2015) | Family Nurse Partnership Programme | Usual care | Biochemically validated 3-day PPA in late pregnancy | Urine cotinine | 243 | 44.42% | 239 | 43.85% | 0.9 | 0.6 | 1.28 | NR |
| Robling (2015) | Family Nurse Partnership Programme | Usual care | Biochemically validated 3-day PPA in late pregnancy (subset of smokers at baseline) | Urine cotinine | 49 | 16.70% | 49 | 16.50% | 1.02 | 0.66 | 1.57 | 0.9414 |
| Robling (2015) | Family Nurse Partnership Programme | Usual care | Biochemically validated 3-day PPA in late pregnancy (subset of non-smokers at baseline) | Urine cotinine | 194 | 76.40% | 180 | 76.60% | 1.22 | 0.82 | 1.83 | 0.3294 |
| Secker-Walker (1997) | Videotape advice | Usual care | Biochemically validated 7-day PPA at the 36th week of pregnancy | CO reading | 5 | 23.81% | 0 | 0.00% | 19 | 0.99 | 365.9 | 0.051 |
| Secker-Walker (1998) 1a | Individual counselling | Usual care | Biochemically validated abstinence at the end of pregnancy | Urine cotinine | 19 | 14.10% | 14 | 9.90% | 1.49 | 0.71 | 3.1 | 0.2909 |
| Secker-Walker (1998) 1b | Individual relapse counselling | Usual care | Biochemically validated abstinence at 36th week of pregnancy (no lapse) | CO reading | 28 | 64.00% | 33 | 69.00% | 0.8 | 0.33 | 1.89 | 0.6045 |
| Secker-Walker (1998) 1b | Individual relapse counselling | Usual care | Biochemically validated abstinence at 36th week of pregnancy | Urine cotinine | 27 | 68.00% | 38 | 81.00% | 0.42 | 0.17 | 1.05 | 0.0642 |
| Stotts (2004) | Motivational interviewing | Usual care | Biochemically confirmed abstinence posttreatment | Urine cotinine | 3 | 21.00% | 8 | 18.00% | 0.77 | 0.16 | 3.64 | 0.7383 |
| Windsor (2000) | SCRIPT procedures | Usual care | Biochemically confirmed 7 day PPA >60 days following first antenatal appointment | Saliva cotinine | 24 | 17.30% | 11 | 8.80% | 2.2 | 2.2 | 4.1 | 0.0439 |
| Windsor (2011) | SCRIPT procedures | Usual care | Biochemically confirmed 7-day PPA >60 days following first antenatal appointment | Saliva cotinine | 65 | 11.95% | 55 | 10.02% | 1.22 | 0.83 | 1.78 | 0.308 |
| Patten (2019) | Biomarker feedback | Quit Tobacco Programme | Self-reported 7-day PPA at week 5 after enrolment | Self-report | 4 | 13.00% | 7 | 23.00% | 0.51 | 0.13 | 1.95 | 0.331 |
| Patten (2019) | Biomarker feedback | Quit Tobacco Programme | Self-reported 7-day PPA at delivery | Self-report | 6 | 20.00% | 7 | 23.00% | 0.82 | 0.24 | 2.81 | 0.7542 |
| Patten (2019) | Biomarker feedback | Quit Tobacco Programme | Biochemically confirmed abstinence at delivery | Urine cotinine | 6 | 20.00% | 6 | 20.00% | 1 | 0.28 | 3.54 | 1 |
| Abroms (2017) | Quit4Baby | Text4Baby | Biochemically confirmed 7-day PPA | Saliva cotinine | 39 | 15.60% | 27 | 10.93% | 1.51 | 0.89 | 2.55 | 0.1269 |
| Abroms (2017) | Quit4Baby | Text4Baby | Biochemically confirmed 30- day PPA | Saliva cotinine | 32 | 12.80% | 26 | 10.53% | 1.19 | 0.69 | 2.07 | 0.5 |
| Abroms (2017) | Quit4Baby | Text4Baby | 7-day self-reported abstinence | Self-report | 72 | 28.80% | 39 | 15.79% | 2.16 | 1.39 | 3.34 | 0.0006 |
| Abroms (2017) | Quit4Baby | Text4Baby | 30-day self-reported abstinence | Self-report | 31 | 12.40% | 19 | 7.69% | 1.7 | 0.93 | 3.1 | 0.08 |
| Abroms (2017) | Quit4Baby | Text4Baby | 7-day self-reported abstinence | Self-report | 88 | 35.20% | 56 | 22.67% | 1.85 | 1.25 | 2.75 | 0.02 |
| Abroms (2017) | Quit4Baby | Text4Baby | 30-day self-reported abstinence | Self-report | 73 | 29.20% | 47 | 19.03% | 1.76 | 1.15 | 2.67 | 0.01 |
| Abroms (2017) | Quit4Baby | Text4Baby | 7-day self-reported abstinence | Self-report | 73 | 29.20% | 63 | 25.51% | 1.2 | 0.81 | 1.79 | 0.36 |
| Abroms (2017) | Quit4Baby | Text4Baby | 30-day self-reported abstinence | Self-report | 60 | 24.00% | 57 | 23.08% | 1.05 | 0.7 | 1.59 | 0.8 |
| Abroms (2017) | Quit4Baby | Text4Baby | 7-day self-reported abstinence in late pregnancy | Self-report | 86 | 34.4.% | 53 | 21.46% | 1.92 | 1.29 | 2.86 | 0.0014 |
| Abroms (2017) | Quit4Baby | Text4Baby | 30-day self-reported abstinence in late pregnancy | Self-report | 69 | 27.60% | 46 | 18.62% | 1.67 | 1.09 | 2.54 | 0.018 |
| Burling (1991) | Letter intervention | Usual care | CO validated smoking cessation at approx. 28th week of pregnancy | CO reading | 8 | 11.60% | 1 | 1.40% | 8.77 | 1.07 | 72.17 | 0.0434 |
| Burling (1991) | Letter intervention | Usual care | CO validated smoking cessation at approx. 34th week of pregnancy | CO reading | 9 | 13.00% | 4 | 5.70% | 2.4 | 0.7 | 8.19 | 0.163 |
| Eades (2012) | Tailored cessation advice and support | Usual care | 7day PPA biochemically validated self-report of abstinence at 36 weeks pregnancy (smokers and spontaneous quitters at baseline) | Urine cotinine | 11 | 7.00% | 4 | 3.00% | 2.23 | 0.69 | 7.2 | 0.1801 |
| Eades (2012) | Tailored cessation advice and support | Usual care | 7day PPA biochemically validated self-report of abstinence at 36 weeks pregnancy (smokers only at baseline) | Urine cotinine | 1 | 1.00% | 2 | 2.00% | 0.43 | 0.04 | 4.77 | 0.4895 |
| Gielen (1997) | Health education and reinforcement | Usual prenatal health education | 7day PPA biochemically validated self-report of abstinence in the third trimester of pregnancy | Saliva cotinine | 12 | 6.20% | 11 | 5.60% | 1.13 | 0.48 | 2.62 | 0.781 |
| Langford (1983) | Prenatal classes with smoking cessation presentations | Control | Self-reported smoking abstinence during pregnancy | Self-report | 17 | 22.10% | 9 | 23.10% | 0.94 | 0.38 | 2.37 | 0.903 |
| Lilley (1986) | Individualised anti-smoking advice | Usual care | Self-reported smoking abstinence | Self-report | 4 | 6.10% | 1 | 1.40% | 4.39 | 0.48 | 40.32 | 0.1914 |
| Lowe (1998) 1b | Healthy '2' booklet | Brief advice | Biochemically validated 7-day PPA at 20 weeks’ gestation | Urine cotinine | 0 | 0.00% | 3 | 8.00% | 5.82 | 0.29 | 116.59 | 0.2495 |
| Pbert (2004) | Smoking cessation 'special' intervention | Usual care | Biochemically validated 7-day PPA in 9th month of pregnancy amongst spontaneous quitters at baseline | Saliva cotinine | NR | NR | NR | NR | 0.95 | 0.37 | 2.45 | 0.9156 |
| Pbert (2004) | Smoking cessation 'special' intervention | Usual care | Biochemically validated 7-day PPA in 9th month of pregnancy amongst smokers at baseline | Saliva cotinine | NR | NR | NR | NR | 2.57 | 1.44 | 4.58 | 0.0014 |
| Price (1991) A/C | Videotape | Physicians’ advice | Smoking cessation in pregnancy | CO reading | 4 | 8.70% | 1 | 4.20% | 2.19 | 0.23 | 20.8 | 0.5 |
| Price (1991) B/C | Self-help booklet | Physicians’ advice | Smoking cessation in pregnancy | CO reading | 2 | 5.10% | 1 | 4.20% | 1.24 | 0.11 | 14.5 | 0.86 |
| Donatelle (2000) | Significant Other Supporter programme | Usual care | Biochemically validated 7-day PPA in 8th month of pregnancy | Saliva cotinine | 37 | 32.00% | 9 | 9.00% | 5.62 | 2.54 | 12.42 | <0.0001 |
| Glover (2015) A/C | Vouchers | Usual care | 7-day PPA for at least 1 week during prenatal period | Self-report | 3 | 37.50% | 1 | 12.50% | 4.2 | 0.33 | 53.13 | 0.2677 |
| Glover (2015) A/C | Vouchers | Usual care | Validated continuous abstinence for 8 weeks | CO reading | 0 | 0.00% | 1 | 12.50% | 0.29 | 0.01 | 8.37 | 0.4737 |
| Glover (2015) B/C | Products | Usual care | 7-day PPA for at least 1 week during prenatal period | Self-report | 6 | 75.00% | 1 | 12.50% | 21 | 1.5 | 293.27 | 0.0236 |
| Glover (2015) B/C | Products | Usual care | Validated continuous abstinence for 8 weeks | CO reading | 2 | 25.00% | 1 | 12.50% | 2.33 | 0.17 | 32.59 | 0.5288 |
| Ondersma (2011) B/D | CM-LITE | Usual care | Biochemically validated abstinence 10 weeks after randomisation | Urine cotinine | 3 | 13.60% | 4 | 17.40% | 0.6 | 0.1 | 4.2 | > .05 |
| Ondersma (2011) B/D | CM-LITE | Usual care | Biochemically validated 7-day PPA 10 weeks after randomisation | CO reading | 2 | 9.10% | 2 | 8.70% | 0.5 | 0.1 | 6.7 | > .05 |
| Ondersma (2011) B/D | CM-LITE | Usual care | Self-reported continuous 30-day abstinence in pregnancy | Self-report | 2 | 9.10% | 1 | 4.30% | 2.7 | 0.2 | 36.6 | > .05 |
| Tuten (2012) A/C | Contingent behavioural incentives | Treatment as usual | Biochemically validated abstinence | Urine cotinine | 13 | 31.00% | 0 | 0.00% | 29.75 | 1.69 | 40 | 0.0203 |
| Tuten (2012) B/C | Non-contingent behavioural incentives | Treatment as usual | Biochemically validated abstinence | Urine cotinine | 0 | 0.00% | 0 | 0.00% | 1.14 | 0.02 | 59.35 | 0.9481 |
| Albrecht (1998) | Teen Freshstart + | TFS and usual care | Biochemically confirmed abstinence 4-6 weeks post randomisation | CO reading | 3 | 11.54% | 5 | 8.62% | 1.38 | 0.3 | 6.28 | 0.6747 |
| Bullock (2008) A/D | Social support plus cessation booklets | Pamphlet | PPA in late pregnancy | Saliva cotinine | 22 | 17.00% | 22 | 17.20% | 0.99 | 0.52 | 1.9 | 0.9774 |
| Bullock (2008) B/D | Social support only | Pamphlet | PPA in late pregnancy | Saliva cotinine | 29 | 22.00% | 22 | 17.20% | 1.36 | 0.73 | 2.51 | 0.3326 |
| Bullock (2008) C/D | Cessation booklets only | Pamphlet | PPA in late pregnancy | Saliva cotinine | 27 | 19.20% | 22 | 17.20% | 1.14 | 0.61 | 2.13 | 0.6773 |
| Hennrikus (2010) | Peer support | Usual care | Biochemically validated 7-day PPA at end of pregnancy | Urine cotinine | 7 | 13.00% | 1 | 3.60% | 2.16 | 0.23 | 20.31 | 0.5006 |
| Malchodi (2003) | Peer counselling | Usual care | Cotinine and CO validated smoking abstinence in the 36th week of pregnancy | Urine cotinine | 16 | 23.88% | 16 | 21.33% | 1.16 | 0.53 | 2.54 | 0.717 |
| Solomon (2000) | Peer phone support | Usual care | Biochemically confirmed 7-day PPA at end of pregnancy | Urine cotinine | 14 | 18.20% | 11 | 14.90% | 1.27 | 0.54 | 3.02 | 0.5841 |
| ***Postnatal smoking abstinence*** | | | | | | | | | | | | |
| Alaniz (2019) | First Breath enhanced programme | First Breath prenatal programme | Validated abstinence | CO reading | 15 | 16.50% | 7 | 7.40% | 2.45 | 0.95 | 6.33 | 0.06 |
| Cinciripini (2010) | CBASP | HW | Self-reported continuous abstinence 6 months post treatment | Self-report | 14 | 11.10% | 11 | 8.50% | 1.3 | 0.6 | 3 | NR |
| Cinciripini (2010) | CBASP | HW | Self-reported 7-day PPA 6 months post treatment | Self-report | 23 | 18.00% | 21 | 16.30% | 1.1 | 0.6 | 2.2 | NR |
| Cinciripini (2010) | CBASP | HW | Self-reported prolonged abstinence 6 months post treatment | Self-report | 18 | 14.10% | 19 | 14.70% | 1 | 0.5 | 2 | NR |
| Cinciripini (2010) | CBASP | HW | Self-reported continuous abstinence 3 months postpartum | Self-report | 15 | 11.70% | 14 | 10.90% | 1.1 | 0.6 | 2 | NR |
| Cinciripini (2010) | CBASP | HW | Self-reported 7-day PPA 3 months postpartum | Self-report | 24 | 18.00% | 23 | 16.30% | 1.1 | 0.5 | 2.4 | NR |
| Cinciripini (2010) | CBASP | HW | Self-reported prolonged abstinence 3 months postpartum | Self-report | 21 | 16.40% | 24 | 18.60% | 0.9 | 0.5 | 1.7 | NR |
| Cinciripini (2010) | CBASP | HW | Self-reported continuous abstinence 6 months postpartum | Self-report | 4 | 3.10% | 3 | 1.20% | 1.7 | 0.4 | 7.3 | NR |
| Cinciripini (2010) | CBASP | HW | Self-reported 7-day PPA 6 months postpartum | Self-report | 9 | 7.00% | 12 | 9.30% | 0.8 | 0.3 | 1.8 | NR |
| Cinciripini (2010) | CBASP | HW | Self-reported prolonged abstinence 6 months postpartum | Self-report | 10 | 7.80% | 8 | 6.20% | 1.3 | 0.5 | 3.4 | NR |
| Coleman-Cowgear (2018) | Phone-based postpartum continuing care | Standard care | Biochemically validated 7-day PPA at 6 weeks postpartum | Urine cotinine |  | 39.00% |  | 25.00% | 1.92 | 0.7 | 5.6 | NS |
| Coleman-Cowgear (2018) | Phone-based postpartum continuing care | Standard care | Biochemically validated 7-day PPA at 3 months postpartum | Urine cotinine |  | 25.00% |  | 14.00% | 2 | 0.6 | 7.3 | NS |
| Coleman-Cowgear (2018) | Phone-based postpartum continuing care | Standard care | Biochemically validated 7-day PPA at 6 months postpartum | Urine cotinine |  | 24.00% |  | 24.00% | 1.04 | 0.3 | 3.3 | NS |
| Dornelas (2006) | Smoking cessation counselling | Usual care | Biochemically validated 7-day PPA 6 months postpartum | CO reading | 5 | 9.40% | 2 | 3.80% | 2.6 | 0.48 | 14.07 | 0.2559 |
| El-Mohandes (2011) | Smoking cessation intervention | Usual care | Biochemically confirmed abstinence 8-10 weeks after delivery | Saliva cotinine | 108 | 55.10% | 85 | 45.21% | 1.49 | 0.99 | 2.22 | 0.0531 |
| Klerman (2011) | Augmented prenatal care | Usual prenatal care | Smoking cessation reported at 1-month postpartum interview | Self-report | 11 | 50.00% | 7 | 26.00% | 2.71 | 0.81 | 9.05 | 0.104 |
| Lee (2015) | Cognitive behavioural counselling | Best practice care | 7-day PPA 1 month postpartum | Saliva cotinine | 21 | 15.00% | 26 | 19.80% | 1.41 | 0.75 | 2.63 | 0.288 |
| Lee (2015) | Cognitive behavioural counselling | Best practice care | 7-day PPA 5 months postpartum | Saliva cotinine | 25 | 17.90% | 16 | 11.70% | 1.63 | 0.82 | 3.2 | 0.155 |
| Mayer (1990) A/C | Behaviour change counselling | Usual care | Smoking abstinence postpartum | Self-report | 5 | 6.94% | 0 | 0.00% | 12.63 | 0.69 | 232.64 | 0.088 |
| Mayer (1990) B/C | Risk information | Usual care | Smoking abstinence postpartum | Self-report | 5 | 7.14% | 0 | 0.00% | 13.02 | 0.71 | 239.81 | 0.0843 |
| Mejdoubi (2014) | Voorzorg Nurse Programme | V-MIS | Smoking cessation at 2 months following birth | Self-report | 141 | 59.49% | 126 | 56.50% | 1.13 | 0.78 | 1.64 | 0.5159 |
| Patten (2020) | Peer counselling with social marketing campaign | Usual care | Self-reported 7-day PPA at 2 months postpartum (all participants at baseline) | Self-report | 56 | 29.79% | 46 | 28.05% | 1.09 | 0.69 | 1.73 | 0.7199 |
| Patten (2020) | Peer counselling with social marketing campaign | Usual care | Self-reported 7-day PPA at 6 months postpartum (all participants at baseline) | Self-report | 52 | 27.66% | 54 | 32.93% | 0.78 | 0.49 | 1.23 | 0.283 |
| Patten (2020) | Peer counselling with social marketing campaign | Usual care | Biochemically validated 7-day PPA at 6 months postpartum (all participants at baseline) | Saliva cotinine | 27 | 14.36% | 29 | 17.68% | 0.78 | 0.44 | 1.38 | 0.3961 |
| Patten (2020) | Peer counselling with social marketing campaign | Usual care | Self-reported 7-day PPA at 2 months postpartum (tobacco users only at baseline) | Self-report | 16 | 13.33% | 11 | 9.65% | 1.44 | 0.64 | 3.25 | 0.3798 |
| Patten (2020) | Peer counselling with social marketing campaign | Usual care | Self-reported 7-day PPA at 6 months postpartum (tobacco users only at baseline) | Self-report | 16 | 13.33% | 17 | 14.91% | 0.88 | 0.42 | 1.83 | 0.7288 |
| Patten (2020) | Peer counselling with social marketing campaign | Usual care | Biochemically validated 7-day PPA at 6 months postpartum (tobacco users only at baseline) | Saliva cotinine | 5 | 4.17% | 3 | 2.63% | 1.61 | 0.38 | 6.89 | 0.5219 |
| Patten (2020) | Peer counselling with social marketing campaign | Usual care | Self-reported 7-day PPA at 2 months postpartum (non- tobacco users only at baseline) | Self-report | 40 | 58.82% | 35 | 70.00% | 0.61 | 0.28 | 1.33 | 0.2141 |
| Patten (2020) | Peer counselling with social marketing campaign | Usual care | Self-reported 7-day PPA at 6 months postpartum (non- tobacco users only at baseline) | Self-report | 38 | 52.94% | 37 | 74.00% | 0.4 | 0.18 | 0.87 | 0.0215 |
| Patten (2020) | Peer counselling with social marketing campaign | Usual care | Biochemically validated 7-day PPA at 6 months postpartum (non- tobacco users only at baseline) | Saliva cotinine | 22 | 32.35% | 26 | 52.00% | 0.44 | 0.21 | 0.94 | 0.0322 |
| Polanska (2005) | Midwife delivered smoking cessation programme | Usual care | Self-reported abstinence 12 months after delivery (spontaneous quitters at baseline) | Self-report | 37 | 69.80% | 23 | 39.70% | 3.52 | 1.6 | 7.74 | 0.0017 |
| Polanska (2005) | Midwife delivered smoking cessation programme | Usual care | Self-reported abstinence 12 months after delivery (smokers at baseline) | Self-report | 28 | 43.70% | 23 | 39.70% | 1.18 | 0.58 | 2.44 | 0.6471 |
| Reitzel (2010) | MAPS/MAPS+ | Usual care | Main treatment effect of quit rates at 8 weeks and 26 weeks postpartum | Saliva cotinine | NR | NR | NR | NR | 1.6 | 1 | 2.58 | 0.05 |
| Reitzel (2010) | MAPS/MAPS+ | Usual care | Biochemically validated continuous abstinence at 8 weeks postpartum | Saliva cotinine | 57 | 41.90% | 32 | 27.80% | 1.87 | 1.1 | 3.18 | 0.0208 |
| Reitzel (2010) | MAPS/MAPS+ | Usual care | Biochemically validated continuous abstinence at 26 weeks postpartum | Saliva cotinine | 31 | 22.80% | 19 | 16.50% | 1.49 | 0.79 | 2.81 | 0.02167 |
| Rigotti (2006) | Smoking cessation counselling | Best practice | Biochemically validated 7-day PPA at 3 months postpartum | Saliva cotinine | 14 | 6.70% | 15 | 7.10% | 0.93 | 0.44 | 1.99 | 1 |
| Rigotti (2006) | Smoking cessation counselling | Best practice | Biochemically validated sustained PPA at 3 months postpartum (at end-of-pregnancy and 3 months postpartum) | Saliva cotinine | 10 | 4.80% | 7 | 3.30% | 1.46 | 0.54 | 3.9 | 0.47 |
| Rigotti (2006) | Smoking cessation counselling | Best practice | Self-report 7-day PPA at 3 months postpartum | Self-report | 24 | 11.50% | 22 | 10.50% | 1.11 | 0.6 | 2.05 | 0.75 |
| Rigotti (2006) | Smoking cessation counselling | Best practice | Self-report sustained PPA at 3 months postpartum (at end-of-pregnancy and 3 months postpartum) | Self-report | 18 | 8.60% | 11 | 5.20% | 1.7 | 0.78 | 3.7 | 0.18 |
| Ruger (2008) | Motivational interviewing | Usual care | Biochemically validated 30-day PPA at 6 months postpartum (smokers at baseline) | Saliva cotinine | 7 | 5.30% | 8 | 7.08% | 0.74 | 0.26 | 2.09 | 0.5644 |
| Ruger (2008) | Motivational interviewing | Usual care | Biochemically validated 30-day PPA at 6 months postpartum (smokers and non-smokers at baseline) | Saliva cotinine | 16 | 10.26% | 13 | 8.90% | 1.17 | 0.54 | 2.52 | 0.6904 |
| Secker-Walker (1998) 1a | Structured advice and individual counselling | Usual care | Self-reported abstinence at 1 year postpartum | Self-report | NR | 11.40% | NR | 6.30% | 1.9 | 0.91 | 4 | NR |
| Secker-Walker (1998) 1b | Structured advice and individual relapse counselling | Usual care | Self-reported abstinence at 1 year postpartum | Self-report | 25 | 55.00% | 32 | 48.00% | 0.76 | 0.36 | 1.57 | 0.4515 |
| Windsor (2011) | SCRIPT procedures | Usual care | Biochemically confirmed 7-day PPA <90 days postpartum | Saliva cotinine | 2 | 0.37% | 4 | 0.73% | 0.5 | 0.09 | 2.76 | 0.4283 |
| Abroms (2017) | Quit4Baby | Text4Baby | 7-day self-reported abstinence postpartum | Self-report | 41 | 16.40% | 42 | 17.00% | 0.96 | 0.6 | 1.53 | 0.8567 |
| Abroms (2017) | Quit4Baby | Text4Baby | 30-day self-reported abstinence postpartum | Self-report | 35 | 14.00% | 38 | 15.38% | 0.9 | 0.54 | 1.47 | 0.6629 |
| Pbert (2004) | Smoking cessation 'special' intervention | Usual care | Biochemically validated 7-day PPA 1 month postpartum amongst smokers at baseline | Saliva cotinine | NR | 26.00% | NR | 11.00% | 3.01 | 1.51 | 5.32 | 0.04 |
| Pbert (2004) | Smoking cessation 'special' intervention | Usual care | Biochemically validated 7-day PPA 3 months postpartum amongst smokers at baseline | Saliva cotinine | NR | 10.00% | NR | 5.00% | 1.91 | 0.68 | 6.17 | 0.65 |
| Strecher (2000) | Tailored smoking cessation guides | Non-tailored cessation guide | Self-reported smoking abstinence at 6 weeks postpartum | Self-report | 10 | 9.60% | 8 | 9.20% | 1.05 | 0.4 | 2.79 | 0.9212 |
| Baker (2018) | Enhanced financial incentives | Routine financial incentives | 1 Week Post birth CO-confirmed 7-day point-prevalence abstinence rates | CO reading | 86 | 17.03% | 68 | 13.36% | 1.33 | 0.94 | 1.88 | 0.1042 |
| Baker (2018) | Enhanced financial incentives | Routine financial incentives | 2-month Post birth CO-confirmed 7-day point-prevalence abstinence rates | CO reading | 87 | 17.23% | 44 | 8.64% | 2.2 | 1.5 | 3.24 | 0.0001 |
| Baker (2018) | Enhanced financial incentives | Routine financial incentives | 3–4-month Post birth CO-confirmed 7-day point-prevalence abstinence rates | CO reading | 85 | 16.83% | 40 | 7.86% | 2.37 | 1.59 | 3.53 | < 0.0001 |
| Baker (2018) | Enhanced financial incentives | Routine financial incentives | 4–6-month Post birth CO-confirmed 7-day point-prevalence abstinence rates | CO reading | 74 | 14.65% | 47 | 9.23% | 1.69 | 1.14 | 2.49 | 0.0083 |
| Baker (2018) | Enhanced financial incentives | Routine financial incentives | 4–6-month Post birth self-reported abstinence rates | Self-report | 81 | 16.00% | 54 | 10.60% | 1.58 | 1.09 | 2.29 | 0.015 |
| Donatelle (2000) | Significant Other Supporter (SOS) programme | Usual care | Biochemically confirmed 7-day PPA 2 months postpartum | Saliva cotinine | 22 | 21.00% | 6 | 6.00% | 4.35 | 1.68 | 11.24 | 0.0024 |
| Bullock (2008) A/D | Social support plus cessation booklets | Pamphlet | PPA 6 weeks post delivery | Saliva cotinine | 16 | 12.40% | 17 | 13.30% | 0.93 | 0.44 | 1.92 | 0.8334 |
| Bullock (2008) B/D | Social support only | Pamphlet | PPA 6 weeks post delivery | Saliva cotinine | 15 | 11.40% | 17 | 13.30% | 0.84 | 0.4 | 1.77 | 0.6383 |
| Bullock (2008) C/D | Cessation booklets only | Pamphlet | PPA 6 weeks post delivery | Saliva cotinine | 19 | 13.50% | 17 | 13.30% | 1.02 | 0.5 | 2.05 | 0.9628 |
| Hennrikus (2010) | Peer support | Usual care | Biochemically validated 7-day PPA 3 months postpartum | Urine cotinine | 5 | 9.30% | 0 | 0.00% | 3.22 | 0.17 | 61.79 | 0.4375 |
| ***Infant birth outcomes*** | | | | | | | | | | | | |
| Mejdoubi (2014) | Voorzorg Nurse Programme | V-MIS | Low birthweight <2500g | Grammes | 25 | 12.30% | 20 | 11.30% | 1.1 | 0.5 | 2.5 | >0.05 |
| Ruger (2008) | Motivational interviewing | Usual care | Low birthweight <2500g | Grammes | 16 | 59.30% | 11 | 40.70% | 2.12 | 0.71 | 6.27 | 0.1761 |
| Secker-Walker (1998) 1a | Structured advice and individual counselling | Usual care | Low birthweight <2500g | Grammes | 7 | 5.20% | 12 | 9.00% | 0.56 | 0.21 | 1.46 | NR |
| Tuten (2012) A/C | Contingent behavioural incentives | Treatment as usual | Low birthweight <2500g | Grammes | 6 | 20.00% | 9 | 42.90% | 0.33 | 0.1 | 1.16 | 0.0834 |
| Tuten (2012) B/C | Non-contingent behavioural incentives | Treatment as usual | Low birthweight <2500g | Grammes | 6 | 37.50% | 9 | 42.90% | 0.73 | 0.19 | 2.72 | 0.6357 |
| Mejdoubi (2014) | Voorzorg Nurse Programme | V-MIS | Preterm gestation (<37 weeks) | Weeks | 16 | 8.60% | 10 | 7.00% | 1.2 | 0.6 | 2.9 | >0.05 |
| Panjari (1999) | Personalised care | Usual care | Preterm delivery <37 weeks | Weeks | 18 | 5.30% | 34 | 8.70% | 1.7 | 0.94 | 3.07 | 0.0789 |
| Tuten (2012) A/C | Contingent behavioural incentives | Treatment as usual | Preterm birth <37 weeks | Weeks | 5 | 16.70% | 15 | 28.60% | 0.08 | 0.02 | 0.3 | 0.0002 |
| Tuten (2012) B/C | Non-contingent behavioural incentives | Treatment as usual | Preterm birth <37 weeks | Weeks | 6 | 35.30% | 15 | 28.60% | 0.22 | 0.05 | 0.86 | 0.0298 |
| **Author (year)** | **Intervention name (I)** | **Control name (C)** | **Outcome description** | **Measure** | **No. (I)** | **Mean (I)** | **SD. (I)** | **No. (C)** | **Mean (C)** | **SD. (C)** | **MD** | **SE** |
| Forinash (2018) | Smoking cessation programme with text messaging | SOC smoking cessation programme | Mean birthweight | Grammes | 11 | 3011.8 | 411.4 | 14 | 3112.8 | 497.9 | -101 | 186.26 |
| Mejdoubi (2014) | Voorzorg Nurse Programme | V-MIS | Mean birthweight difference | Grammes | 217 | 3144 | 577 | 188 | 3147 | 519 | -3 | 54.884 |
| Ruger (2008) | Motivational interviewing | Usual care | Mean infant birthweight | Grammes | 131 | 3241.2 | 586 | 128 | 3321.3 | 612.1 | -80.1 | 74.45 |
| Panjari (1999) | Personalised care | Usual care | Mean birthweight (all births) | Grammes | 337 | 3250 | 526 | 391 | 3166 | 589 | 84 | 41.678 |
| Robling (2015) | Family Nurse Partnership Programme | Usual care | Mean birthweight | Grammes | 742 | 3217.4 | 618 | 768 | 3197.5 | 581.5 | 19.9 | 30.871 |
| Secker-Walker (1998) | Structured advice and individual counselling | Usual care | Mean birthweight | Grammes | NR | 3256 | 452 | NR | 3221 | 506 | 35 | 58.491 |
| Tuten (2012) A/C | Contingent behavioural incentives | Treatment as usual | Mean birthweight | Grammes | 30 | 2863.3 | 694.3 | 21 | 2701.3 | 598.3 | 162 | 186.877 |
| Tuten (2012) B/C | Non-contingent behavioural incentives | Treatment as usual | Mean birthweight | Grammes | 17 | 2695.6 | 656.9 | 21 | 2701.3 | 598.3 | -5.43 | 203.917 |
| Forinash (2018) | Smoking cessation programme with text messaging | SOC smoking cessation programme | Mean 5-minute APGAR | Count | 11 | 8.25 | 1 | 14 | 8.33 | 1.6 | -0.08 | 0.533 |
| Tuten (2012) A/C | Contingent behavioural incentives | Treatment as usual | Mean AGPAR 5-minute score | Count | 30 | 8.7 | 1.7 | 21 | 8.4 | 1.3 | 0.3 | 0.441 |
| Tuten (2012) B/C | Non-contingent behavioural incentives | Treatment as usual | Mean AGPAR 5-minute score | Count | 17 | 8.8 | 0.6 | 21 | 8.4 | 1.3 | 0.4 | 0.342 |

*No.=number, prop= proportion, PPA = point prevalence abstinence, CO = carbon monoxide, SD = standard deviation, MD = mean difference, SE = standard error*
