# Supplementary material for: Are psychosocial smoking cessation interventions delivered in pregnancy equally effective? A systematic review, meta-analysis and equity analysis of moderation analyses in randomized controlled trials
Source: J Behav Med. 2025 Nov 16;49(1):1–14. doi: 10.1007/s10865-025-00614-6 (PMC12945952; doi:10.1007/s10865-025-00614-6)
Supplement: Supplementary file 3 — Supplementary file3 (DOCX 2027 kb) [file 10865_2025_614_MOESM3_ESM.docx]

Appendix 3 – additional analyses

Table of Contents

[Forest plots 2](#_Toc171002334)

[Risk of bias assessments 6](#_Toc171002335)

[Funnel plots 9](#_Toc171002336)

[GRADE assessments 10](#_Toc171002337)

[Meta-regressions 11](#_Toc171002338)

# **Forest plots**

**
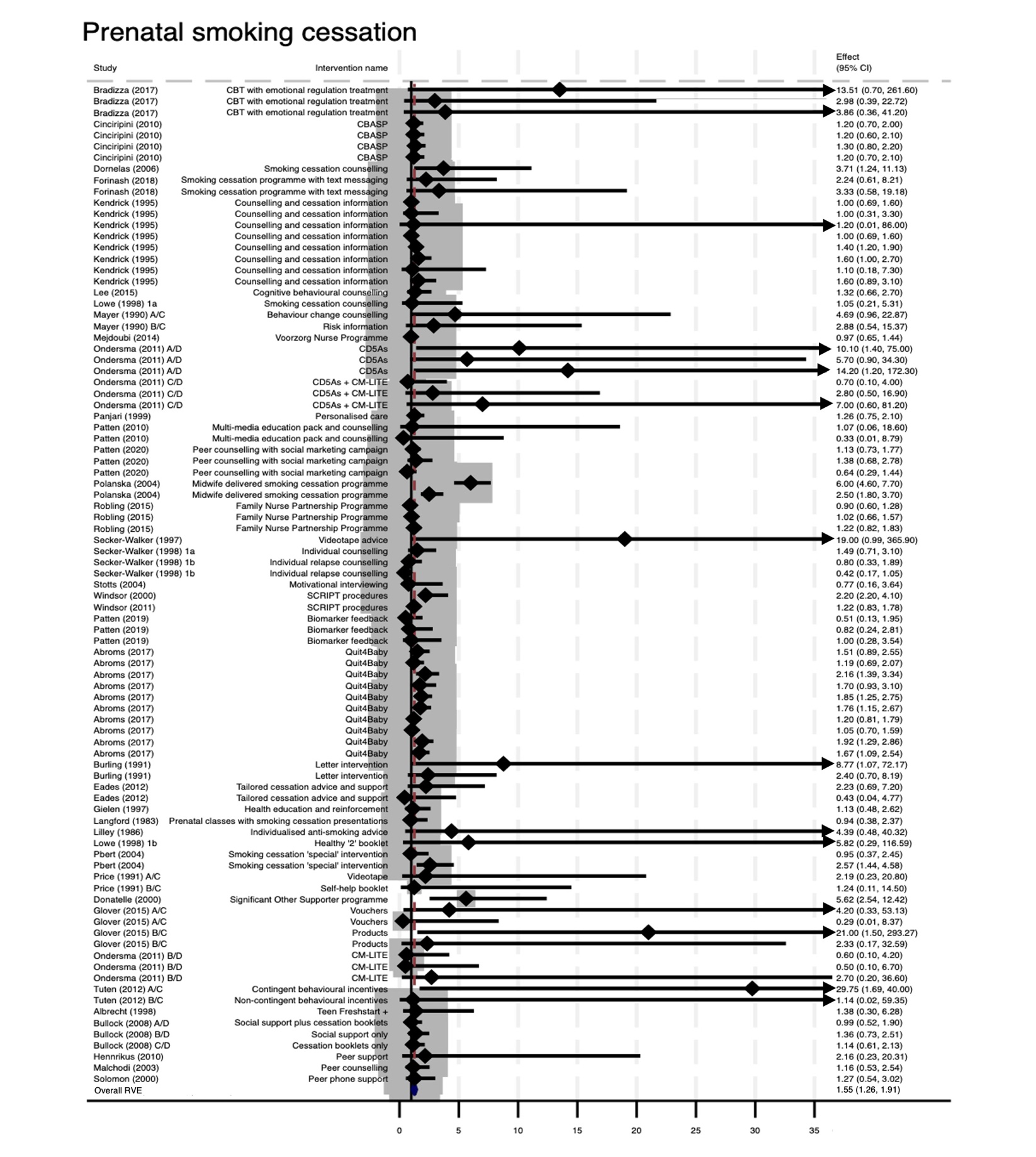
**Appendix 3 figure 1 – prenatal smoking cessation meta-analysis

Appendix 3 figure 2 – postnatal smoking abstinence meta-analysis


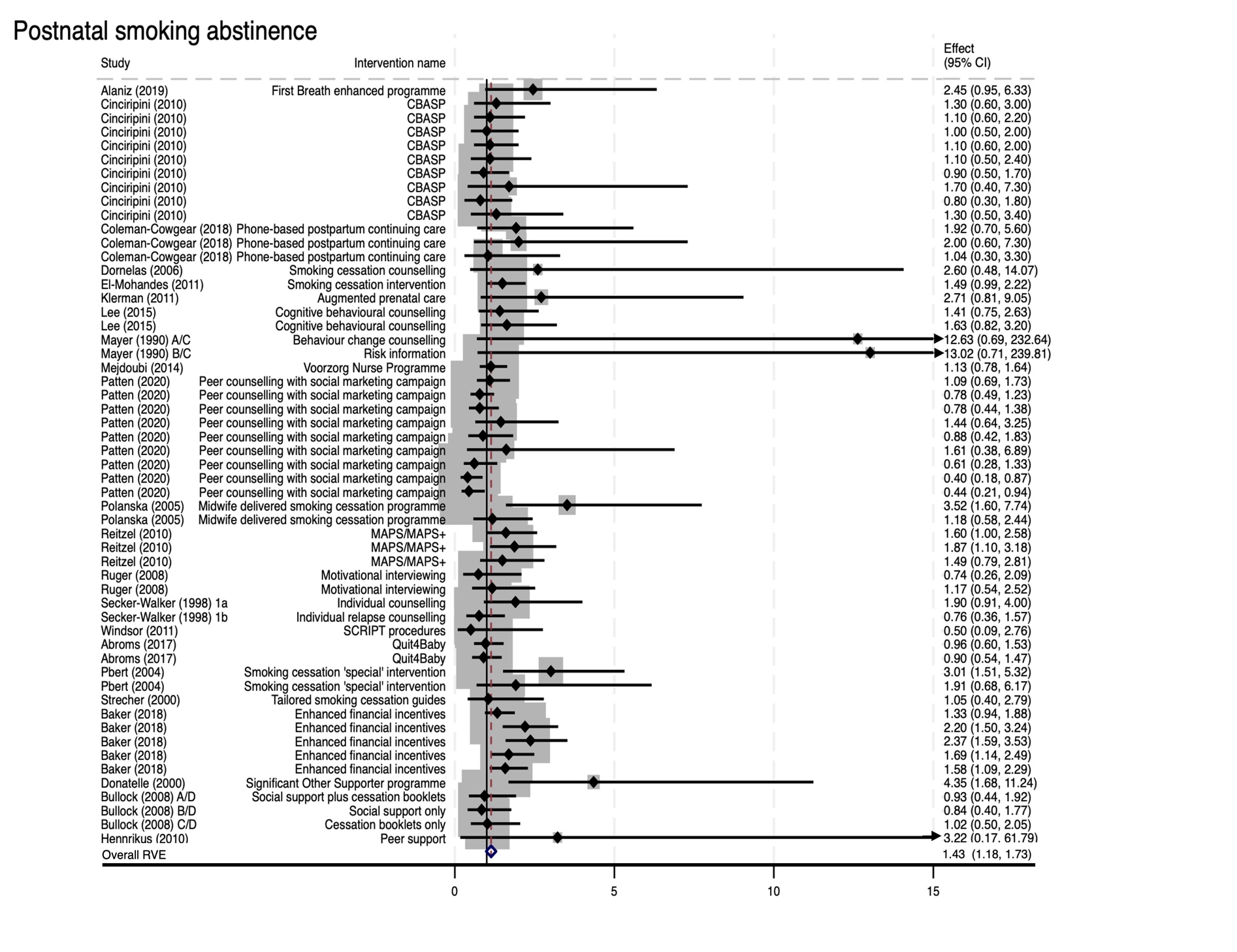


Appendix 3 figure 3 – mean difference birthweight meta-analysis
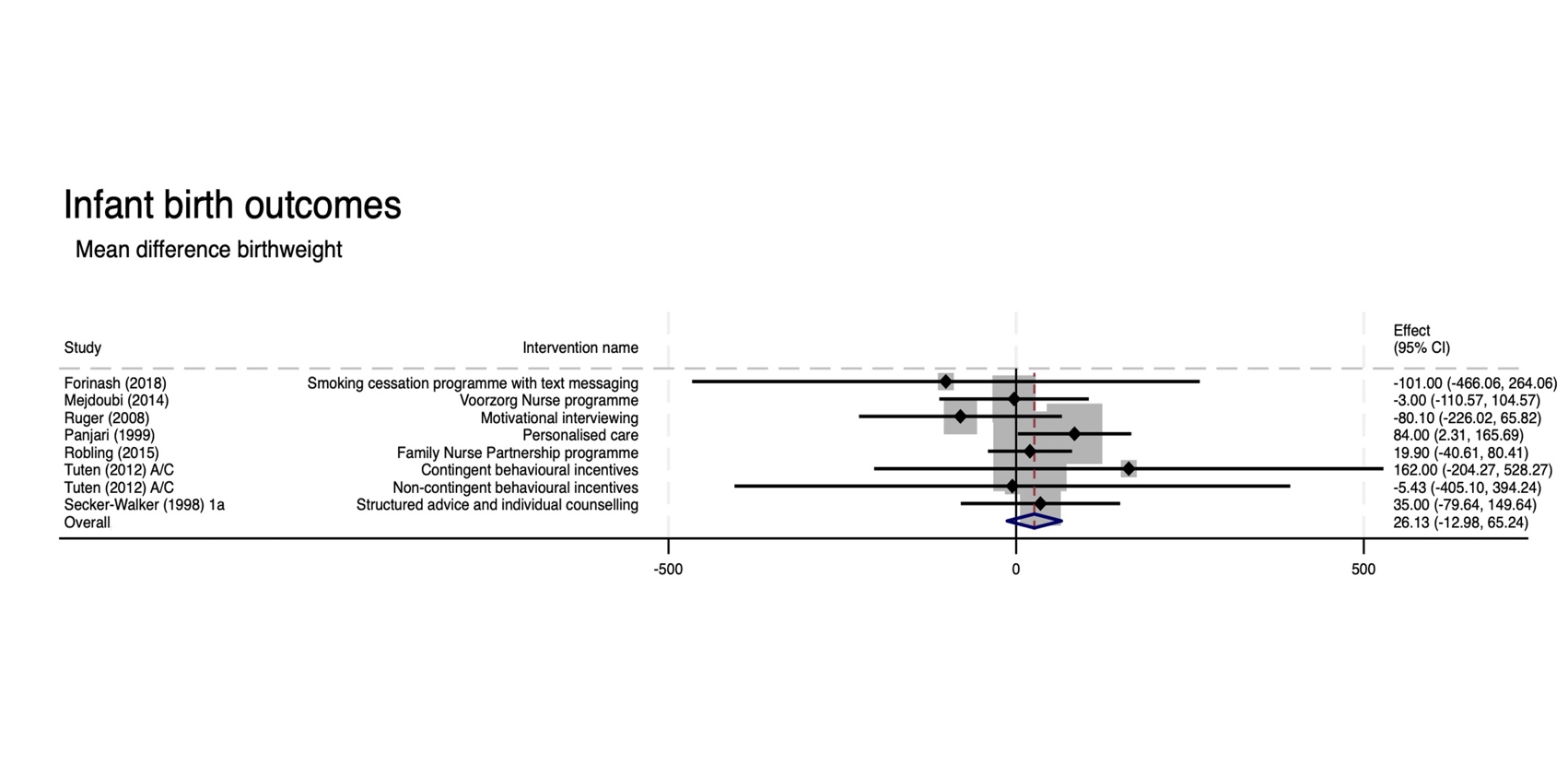


Appendix 3 figure 4 – mean APGAR meta-analysis
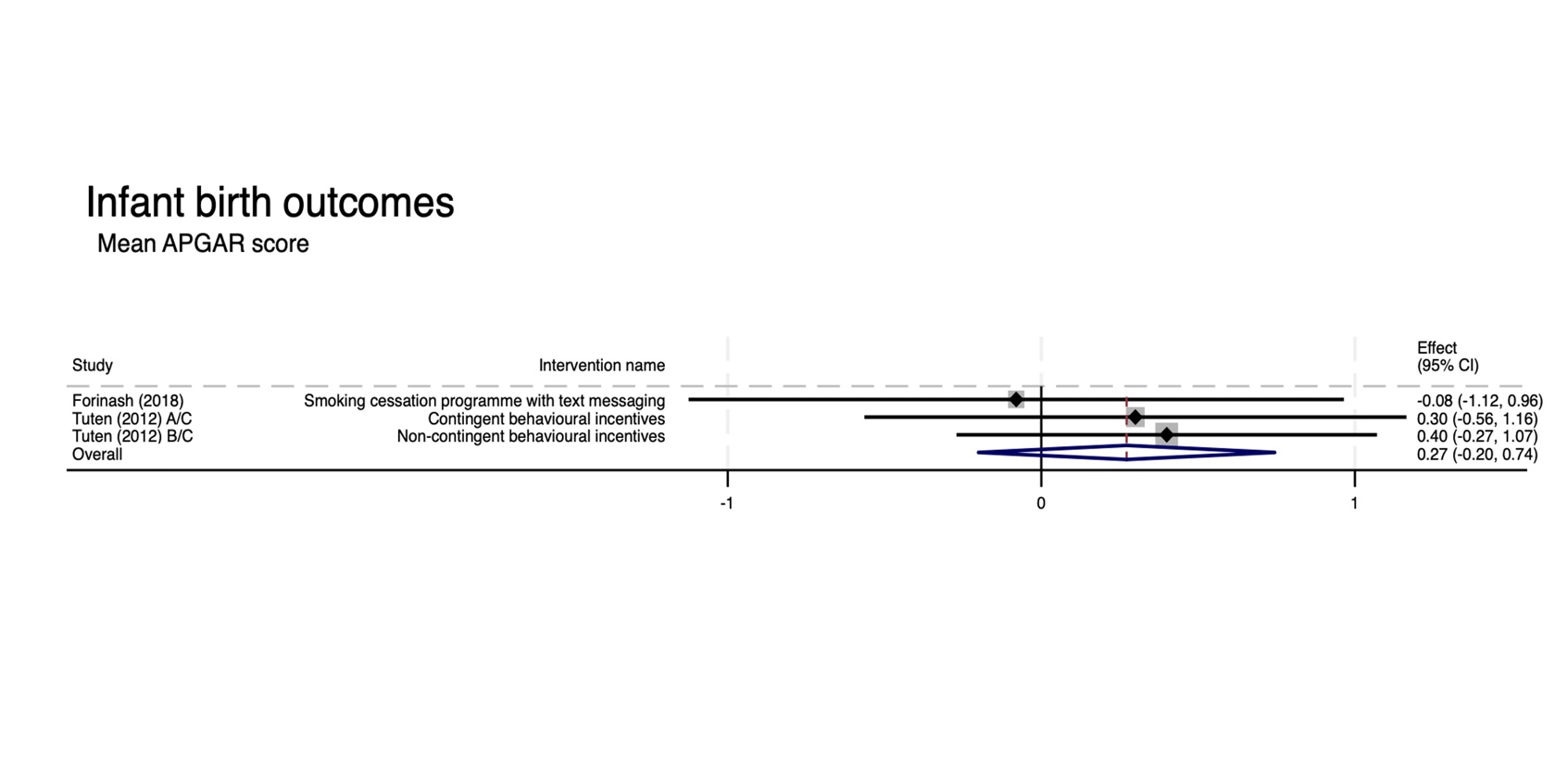


Appendix 3 figure 5 – low birthweight meta-analysis
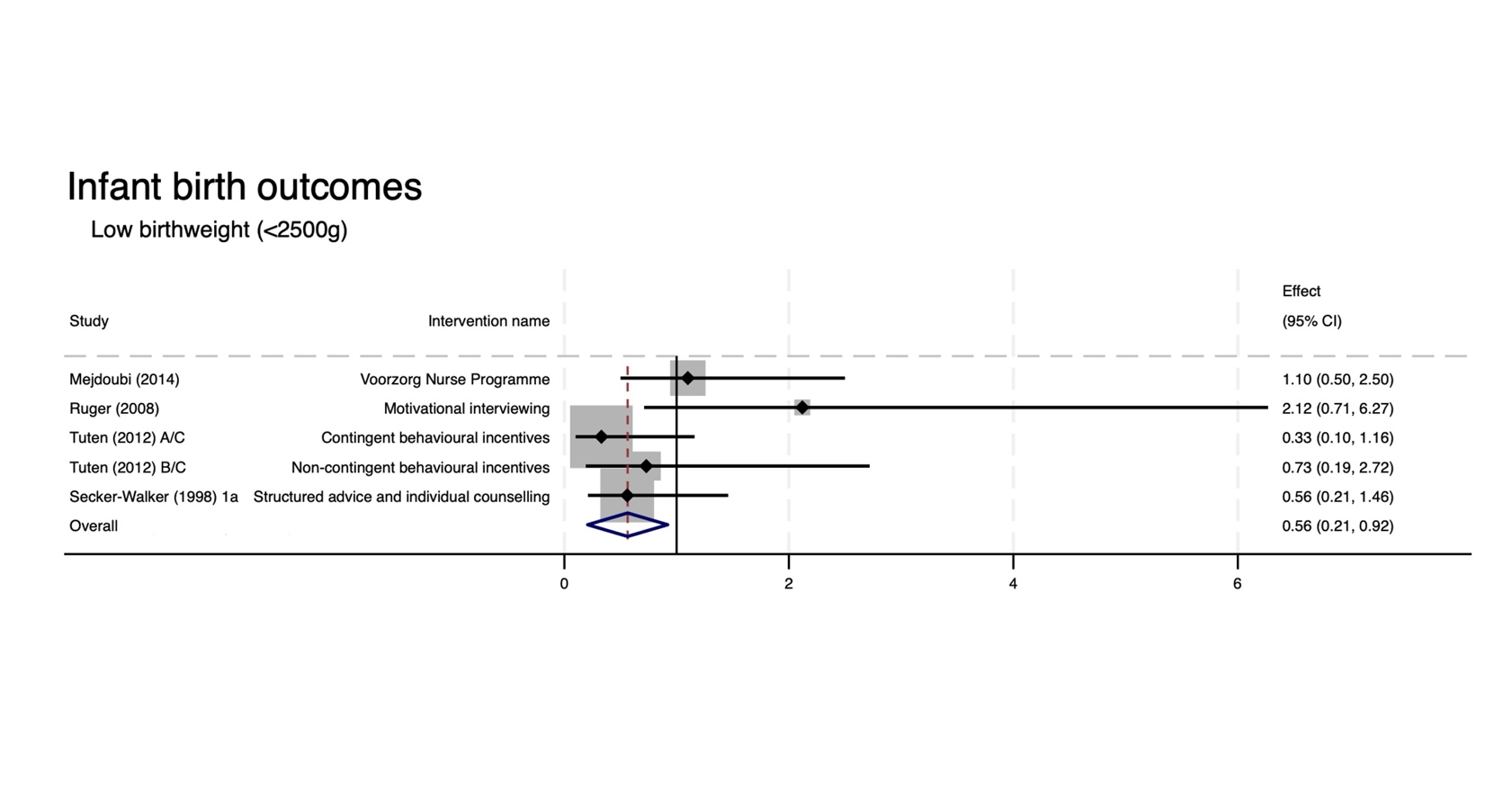


Appendix 3 figure 6 – preterm birth meta-analysis


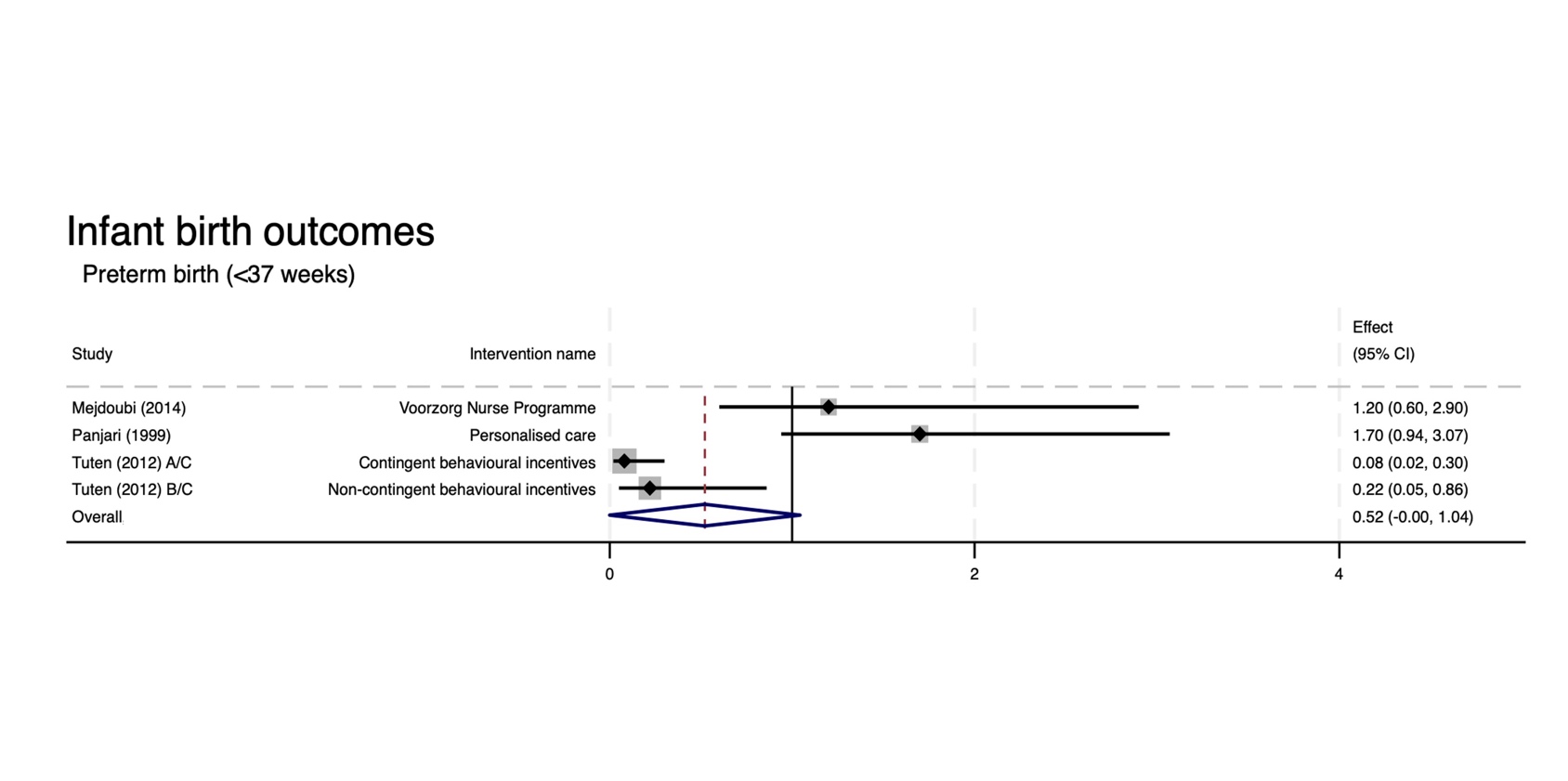


# **Risk of bias assessments**

Appendix 3 table 1 - randomised controlled trials (smoking outcomes):

| **Study** | **1: Randomisation process** | **2: Deviation from intended intervention** | **3: Missing outcome data** | **4: Measurement of the outcome** | **5: Selection of the reported result** | **6: Overall risk of bias** |
| --- | --- | --- | --- | --- | --- | --- |
| Abroms, 2017 | Low | Low | Low | Low | Low | Low |
| Ershoff, 1999 | Some concerns | High | Low | Low | Some concerns | High |
| Forinash, 2018 | Low | High | High | Low | Low | High |
| Windsor, 1993 | Low | Some concerns | Low | Low | Some concerns | Some concerns |
| Alaniz, 2019 | Some concerns | Some concerns | High | Low | Some concerns | High |
| Baker, 2018 | Some concerns | Low | High | Low | Some concerns | High |
| Bullock, 2008 | Low | Low | Low | Low | Some concerns | Some concerns |
| Coleman-Cowgear, 2018 | Low | Some concerns | Low | Low | Some concerns | Some concerns |
| Eades, 2012 | High | Low | Low | Low | Low | High |
| Gielen 1997 | Some concerns | Low | Some concerns | Low | Some concerns | Some concerns |
| Glover, 2015 | Low | Some concerns | High | Low | Low | High |
| Klernan, 2011 | Low | Some concerns | Low | High | Some concerns | High |
| Lee, 2015 | Some concerns | Low | Low | Low | Low | Some concerns |
| Lowe, 1998 1a | Some concerns | Some concerns | High | Low | Some concerns | High |
| Lowe, 1998 1b | Some concerns | Some concerns | Low | Low | Some concerns | Some concerns |
| Mejdoubi, 2014 | Low | Some concerns | Low | High | Some concerns | High |
| Patten, 2019 | Some concerns | Some concerns | Low | Low | Some concerns | Some concerns |
| Patten, 2010 | Some concerns | Some concerns | Some concerns | Low | Low | Some concerns |
| Price, 1991 | Low | High | High | Low | Some concerns | High |
| Ruger, 2008 | Some concerns | Some concerns | Some concerns | Low | Some concerns | Some concerns |
| Tappin, 2022 | Low | Low | Low | Low | Low | Low |
| Windsor, 2011 | Some concerns | Some concerns | Some concerns | Low | Some concerns | Some concerns |
| Windsor, 2000 | High | Some concerns | Some concerns | Some concerns | Some concerns | High |
| Stretcher, 2000 | Low | Some concerns | Low | Some concerns | Some concerns | Some concerns |
| Donatelle, 2000 | Some concerns | Some concerns | Some concerns | Low | Some concerns | Some concerns |
| Langford, 1983 | Some concerns | Some concerns | High | High | Some concerns | High |
| Mayer, 1990 | Some concerns | Some concerns | Some concerns | High | Some concerns | High |
| Panjari, 1999 | Some concerns | Some concerns | Some concerns | Low | Some concerns | Some concerns |
| Malchodi, 2003 | Low | Some concerns | Low | Low | Some concerns | Some concerns |
| Burling, 1991 | Some concerns | Some concerns | Low | Low | Some concerns | Some concerns |
| Lilley, 1986 | Some concerns | Some concerns | Low | High | Some concerns | High |
| Solomon, 2000 | Some concerns | Some concerns | Low | Low | Some concerns | Some concerns |
| Robling, 2015 | Low | Some concerns | Some concerns | Low | Low | Some concerns |
| Bradizza, 2017 | Some concerns | Some concerns | Low | Low | Some concerns | Some concerns |
| Dornelas, 2006 | Some concerns | Some concerns | Low | Low | Some concerns | Some concerns |
| Secker-Walker 1997 | Some concerns | High | Low | Low | Some concerns | High |
| Hennrikus, 2010 | Some concerns | Some concerns | Some concerns | Low | Some concerns | Some concerns |
| Tuten, 2012 | Some concerns | Some concerns | Low | Low | Some concerns | Some concerns |
| Brandon, 2012 | Some concerns | Some concerns | Low | High | Some concerns | High |
| Secker-Walker, 1998 1a | Some concerns | Some concerns | Low | Low | Some concerns | Some concerns |
| Secker-Walker, 1998 1b | Some concerns | Some concerns | Low | Low | Some concerns | High |
| Reitzel, 2010 | Low | Some concerns | Low | Low | Some concerns | Some concerns |
| Ondersma, 2011 | Low | Some concerns | Low | Low | Low | Some concerns |
| Albrecht, 1998 | Some concerns | High | Some concerns | Low | Some concerns | High |
| Stotts, 2004 | Some concerns | High | High | Low | Some concerns | High |
| El-Mohandes, 2011 | Low | Some concerns | Low | Low | Some concerns | Some concerns |
| Rigotti, 2006 | Low | Some concerns | Low | Low | Some concerns | Some concerns |
| Cinciripini, 2010 | Some concerns | Some concerns | Some concerns | Low | Some concerns | Some concerns |

Appendix 3 table 2 - cluster randomised controlled trials (smoking outcomes):

| **Study** | **1a: Randomisation process** | **1b: Timing of recruitment of participants** | **2: Deviation from intended intervention** | **3: Missing outcome data** | **4: Measurement of the outcome** | **5: Selection of the reported result** | **6: Overall risk of bias** |
| --- | --- | --- | --- | --- | --- | --- | --- |
| Pbert (2004) | Some concerns | Some concerns | Some concerns | Low | Low | Some concerns | Some concerns |
| Polanska (2004) | Some concerns | Some concerns | Some concerns | Low | High | Some concerns | High |
| Polanska (2005) | Some concerns | Some concerns | Some concerns | Low | High | Some concerns | High |
| Patten (2020) | Low | High | Some concerns | Low | High | Low | High |
| Hajek (2001) | Low | High | Some concerns | Low | Low | Some concerns | High |
| Kendrick (1995) | Some concerns | Some concerns | Some concerns | High | Low | Some concerns | High |

Appendix 3 table 3 - randomised controlled trials (infant birth outcomes):

| **Study** | **1 : Randomisation process** | **2 : Deviation from intended intervention** | **3 : Missing outcome data** | **4 : Measurement of the outcome** | **5 : Selection of the reported result** | **6 : Overall risk of bias** |
| --- | --- | --- | --- | --- | --- | --- |
| Forinash, 2018 | Low | High | High | Some concerns | Low | High |
| Mejdoubi, 2014 | Low | Some concerns | Low | Low | Some concerns | Some concerns |
| Ruger, 2008 | Some concerns | Some concerns | Low | Low | Some concerns | Some concerns |
| Tuten, 2012 | Some concerns | Some concerns | Low | Low | Some concerns | Some concerns |
| Secker-Walker, 1998 | Some concerns | Some concerns | Low | Low | Some concerns | Some concerns |
| Panjari, 1999 | Some concerns | Some concerns | Low | Low | Some concerns | Some concerns |
| Hebel, 1985 | Some concerns | Some concerns | Low | Low | Some concerns | Some concerns |

# **Funnel plots**

Funnel plots include all active interventions compared to control. The effect size is plotted on the log odds scale. Contours represent pseudo 90%, 95% and 99% confidence intervals. Funnel plots were not produced for infant birth outcomes which had included less than 10 studies.

Appendix 3 figure 7 – funnel plot for prenatal smoking cessation


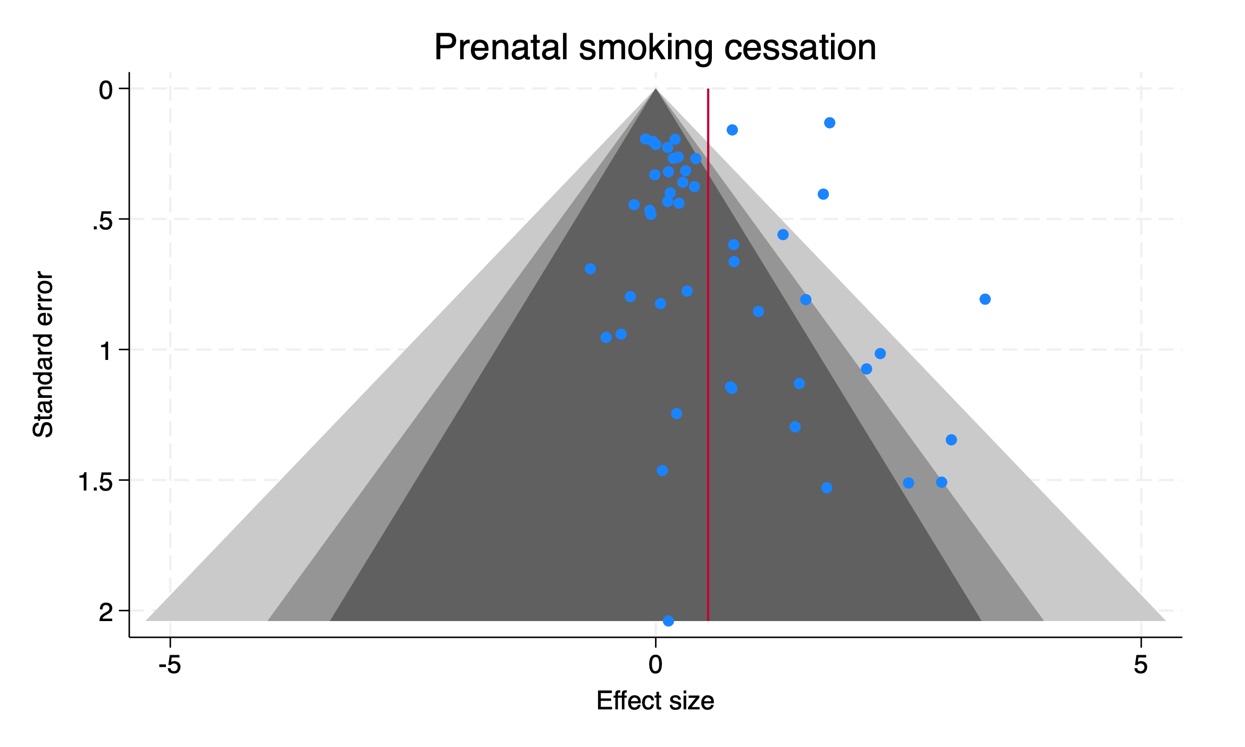


Appendix 3 figure 8 – funnel plot for postnatal smoking abstinence

**
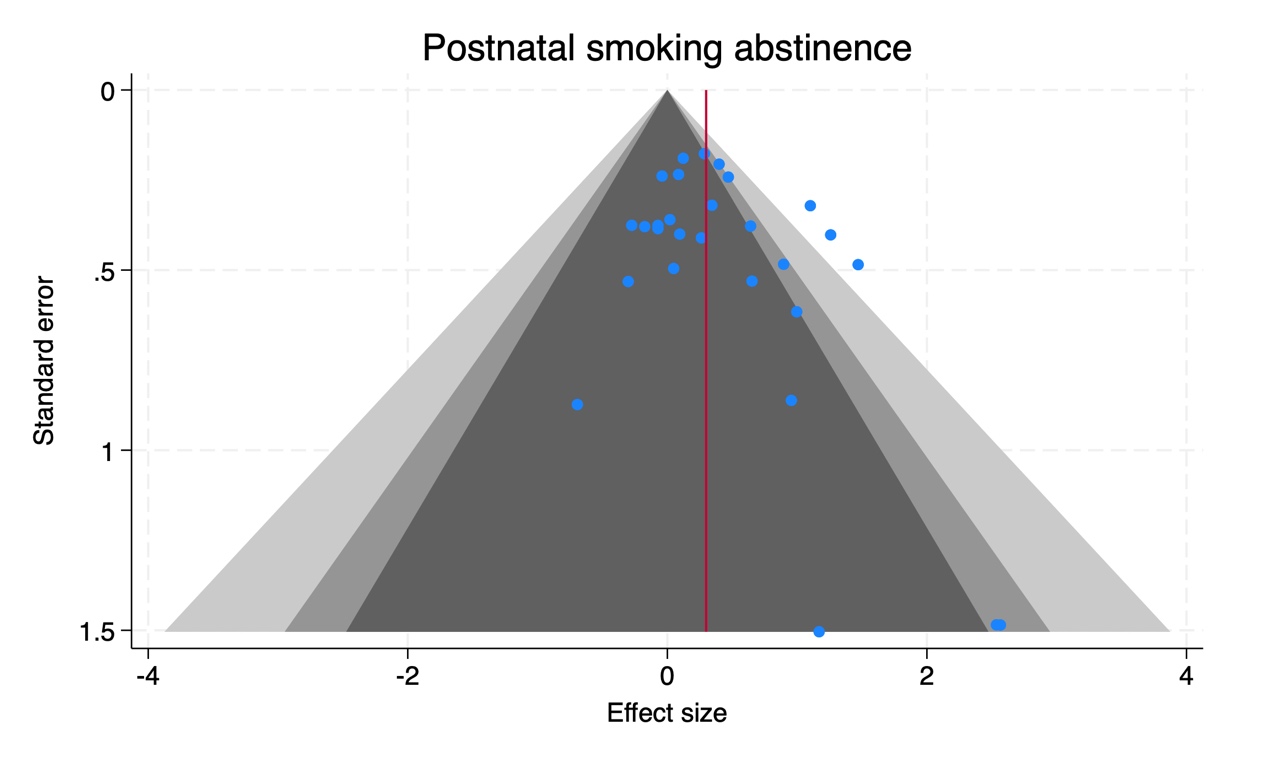
**

# **Quality assessments**

| **Certainty assessment** | | | | | | | **Impact** | **Certainty** |
| --- | --- | --- | --- | --- | --- | --- | --- | --- |
| **№ of studies** | **Study design** | **Risk of bias** | **Inconsistency** | **Indirectness** | **Imprecision** | **Other considerations** |  |  |
| **Prenatal smoking cessation** | | | | | | | | |
| 38 | randomised trials | serious | serious | not serious | not serious | publication bias suspected | OR 1.57  (1.26,1.94) | ⨁◯◯◯  Very low |
| **Postnatal smoking abstinence** | | | | | | | | |
| 21 | randomised trials | serious | serious | not serious | not serious |  | OR 1.43 (1.19, 1.73) | ⨁⨁◯◯  Low |
| **Infant birthweight** | | | | | | | | |
| 7 | randomised trials | not serious | not serious | not serious | not serious |  | MD 26.13  (-12.98, 65.24) | ⨁⨁⨁◯  Moderate |
| **Infant APGAR score** | | | | | | | | |
| 2 | randomised trials | not serious | not serious | not serious | not serious |  | MD 0.22  (-0.16, 0.59) | ⨁⨁⨁◯  Moderate |
| **Infant low birthweight** | | | | | | | | |
| 4 | randomised trials | not serious | not serious | not serious | not serious |  | OR 0.56  (0.21, 0.92) | ⨁⨁⨁◯  Moderate |
| **Preterm birth** | | | | | | | | |
| 3 | randomised trials | not serious | not serious * | not serious | not serious |  | OR 0.52  (0, 1.04) | ⨁⨁⨁◯  Moderate |

Appendix 3 table 5 – GRADE assessment

** between study differences in effect sizes likely explained by different populations*

# **Meta-regressions for prenatal smoking cessation outcome**

Proportion of minoritised ethnic participants in the trial sample was added as a continuous variable and as a binary variable (comparing trials with >80% minoritised participants and those without) for robustness.

Appendix 3 table 6 – meta-regressions by proportion of minoritised ethnic participants

| **Analysis** | **Term** | **OR (95% CI)** |
| --- | --- | --- |
| Proportion of ethnic minoritised participants (using continuous variable) | Intercept | 1.79 (1.08, 2.98) |
|  | Coefficient | 0.74 (0.36, 1.54) |
| Proportion of ethnic minoritised participants (using binary variable) | Intercept | 1.63 (1.27, 2.10) |
|  | Coefficient | 0.81 (0.43, 1.52) |
